# Gene dysregulation is restored in the Parkinson’s disease MPTP neurotoxic mice model upon treatment of the therapeutic drug Cu^II^(atsm)

**DOI:** 10.1038/srep22398

**Published:** 2016-03-01

**Authors:** Lesley Cheng, Camelia Y. J. Quek, Lin W. Hung, Robyn A. Sharples, Nicki A. Sherratt, Kevin J. Barnham, Andrew F. Hill

**Affiliations:** 1 Department of Biochemistry and Molecular Biology, The University of Melbourne, Melbourne, Victoria, 3010, Australia; 2 Department of Pharmacology and Therapeutics, The University of Melbourne, Melbourne, Victoria, 3010, Australia; 3 Bio21 Molecular Science and Biotechnology Institute, The University of Melbourne, Victoria, 3010, Australia; 4 The Florey Institute of Neuroscience and Mental Health, The University of Melbourne, Melbourne, Victoria 3010, Australia; 5 Department of Biochemistry and Genetics, La Trobe Institute for Molecular Science, La Trobe University, Bundoora, Victoria 3086, Australia

## Abstract

The administration of MPTP selectively targets the dopaminergic system resulting in Parkinsonism-like symptoms and is commonly used as a mice model of Parkinson’s disease. We previously demonstrated that the neuroprotective compound Cu^II^(atsm) rescues nigral cell loss and improves dopamine metabolism in the MPTP model. The mechanism of action of Cu^II^(atsm) needs to be further defined to understand how the compound promotes neuronal survival. Whole genome transcriptomic profiling has become a popular method to examine the relationship between gene expression and function. Substantia nigra samples from MPTP-lesioned mice were evaluated using whole transcriptome sequencing to investigate the genes altered upon Cu^II^(atsm) treatment. We identified 143 genes affected by MPTP lesioning that are associated with biological processes related to brain and cognitive development, dopamine synthesis and perturbed synaptic neurotransmission. Upon Cu^II^(atsm) treatment, the expression of 40 genes involved in promoting dopamine synthesis, calcium signaling and synaptic plasticity were restored which were validated by qRT-PCR. The study provides the first detailed whole transcriptomic analysis of pathways involved in MPTP-induced Parkinsonism. In addition, we identify key therapeutic pathways targeted by a potentially new class of neuroprotective agents which may provide therapeutic benefits for other neurodegenerative disorders.

Parkinson’s disease (PD) is characterised by the presence of Lewy bodies, increased synucleinopathy, dopamine reduction in the striatum and degeneration of dopaminergic neurons in the substantia nigra pars compacta (SNpc). The consequence of such disease leads to motor impairments, including tremor dyskinesia, rigidity, instability and tremors^1^. The major pathological hallmarks include mitochondrial impairment, oxidative stress and neuroinflammation leading to neuronal cell death.

Neurotoxin models of PD are widely used and often require a pathological lesion that results in an approximate 50% decrease of dopaminergic neurones in the SNpc to produce the phenotypic features of late-stage PD. One of the most commonly used neurotoxin based animal models to study PD is the 1-methyl-1, 2, 3, 6-tetrahydropyidine (MPTP) induced mice model^2^,^3^,^4^,^5^,^6^. Traditional biochemical methods have permitted researchers to explore the molecular functions and dissect the pathways implicated during neurodegeneration of dopaminergic neurons in the MPTP-induced models. Upon intraperitoneal injection of MPTP, the neurotoxin crosses the blood brain barrier (BBB) and is subsequently converted to 1-methyl-4-phenylpyridinium ion (MPP^+^) by glial monoamine oxidase B. MPP^+^ is a substrate of the dopamine transporter. MPP^+^ then enters the mitochondria and disrupts oxidative phosphorylation by inhibiting complex I of the mitochondrial electron transport chain and results in a mitochondrial-initiated mode of cell death^7^,^8^. Molecular approaches using microarrays have shown various changes in gene expression observed in MPTP models, such as, an increase in Bax messenger RNA (mRNA)^8^,^9^. In addition, a number of PD associated genes (*Th;* tyrosine hydroxylase, *Nd4;* NADH dehydrogenase, subunit 4, *Sncb;* β-synuclein, *Uchl1;* ubiquitin carboxyl-terminal esterase L1, *Hsp70;* heat shock protein 70, *Park2;* E3 ubiquitin protein ligase, *NFkB*; nuclear factor kB and *iNOS*; inducible nitric oxide synthase) are found to be altered at various time-points during the course of MPTP lesioning^10^,^11^. Investigating gene alterations in the MPTP mice model that may be involved in the etiology of PD is an approach to identify new potential neuroprotective targets, therapeutic targets and biomarkers^12^.

Several studies have used the MPTP model to demonstrate the effectiveness of potential neuroprotective drugs for PD such as the iron chelator, M30, and others including ladostigil and TV-3326 (N-propargyl-3R-aminoindan-5yl)-ethyl methylcarbamate) which have been found to regulate a number of neuroprotective-adaptive mechanisms^13^,^14^. Previously, we identified a potential therapeutic for PD, copper(II)diacetylbis(N(4)-methylthiosemicarbazonato (Cu^II^(atsm)), which rescued PD-relevant phenotypes in multiple animal models including the MPTP mice model^2^. The administration of MPTP in C57BL/6 mice (40 mg/kg i.p.) caused significant reduction (50%; P < 0.001) in the number of dopaminergic neurons within the SNpc which caused impairment of motor function and decrease of *Th* and dopamine metabolism^2^. Cu^II^(atsm) treatment was able to rescue nigral cell loss and improve dopamine metabolism in the MPTP model. The potential mechanism behind the neuroprotective affect of Cu^II^(atsm) is the ability of this compound to inhibit peroxynitrate-mediated nitrative stress and consequently the formation of α-synuclein aggregates. It is possible that Cu^II^(atsm) acts on other pathways to promote neuronal survival which needs to be investigated.

Until now, studies have employed the use of qRT-PCR and microarray technology to decipher the molecular mechanism behind potential therapeutics for PD within mice models. Here, we have employed an RNAseq whole transcriptomic sequencing approach to evaluate the SNpc from MPTP lesioned mice and the changes upon Cu^II^(atsm) treatment. This unbiased high-throughput approach enables the identification of genes relevant to PD that have not been previously associated with the disease and the cellular genes and pathways targeted by Cu^II^(atsm). Furthermore, in addition to Cu^II^(atsm), new targets could be identified to assist in the development of neuroprotective drugs that may exert their protective effect through their ability to modulate target gene expression. In our previous study, MPTP induced decreases in *Snca, Th* and *Vmat2* (vesicular monoamine transporter 2) mRNA expression which recovered to normal levels upon treatment with Cu^II^(atsm). Our previous study provides a rationale to identify all deregulated genes in MPTP-lesioned mice to understand the pathways targeted by this compound 2.

Here, we identified significantly altered gene expression in targets associated with perturbed synaptic neurotransmission, stress response, Mitogen-activated protein kinases (MAPK) signalling, cell adhesion, nervous system development and vascular smooth muscle contraction (VSMC) in MPTP-lesioned mice. Additional intrinsic signalling pathways in respect to activated calcium signalling, phosphatidylinositol 3′ –kinase—protein kinase B (PI3k-AKT) signalling, and neuronal junctions were also identified and were found to recover upon Cu^II^(atsm) treatment. This study unveils a consistent pattern of expression changes in the transcriptome that correlates with injured or dying neurons under neurotoxic stress by acquiring either adaptive, compensatory or restoratory molecular responses.

## Results

### Distribution of gene expression in SNpc of MPTP-lesioned mice

**Figure 1:**
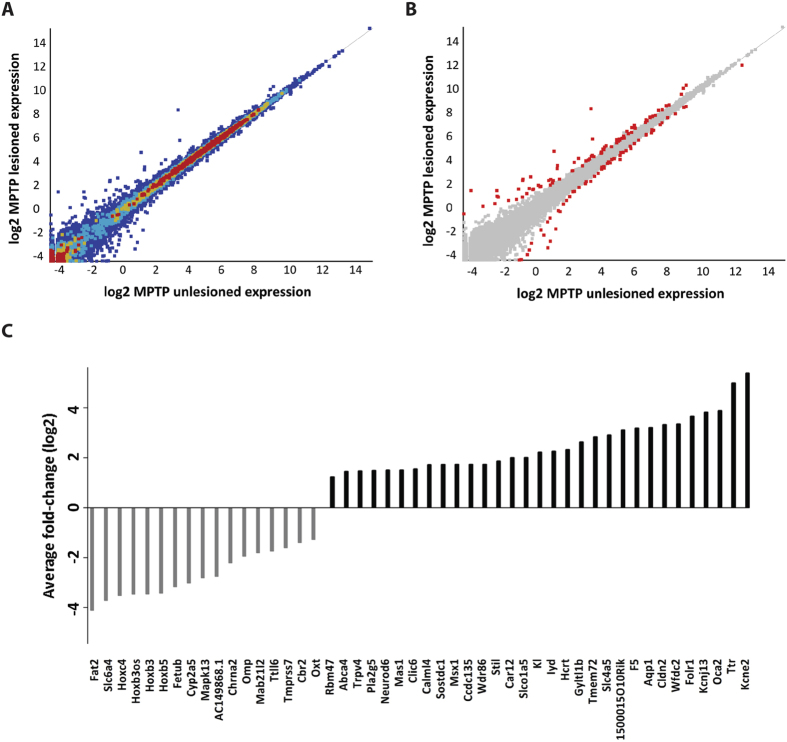
Gene expression distribution between unlesioned and MPTP-lesioned samples. (**A**) A total of 25,900 genes were identified in the samples and plotted. The colours represent different densities of genes in which red colour denote a high number of genes are overlaid at a particular position and blue colour denotes otherwise. (**B**) Scatter plot highlights 143 significantly DE genes between unlesioned and MPTP-lesioned samples (P < 0.05 with Benjamini-Hochberg multiple testing correction) (red data-points). (**C**) DE genes with significant fold change. Genes identified with a greater than 1.2 fold change (log2) and p-value < 0.05 (cut off at 5% FDR) are displayed here. Unlesioned (n = 6), MPTP lesioned (n = 5), MPTP lesioned mice treated with Cu^II^(atsm) (n = 7) and unlesioned control treated with Cu^II^(atsm) (n = 4).

Upon whole transcriptome RNA deep sequencing, the bioinformatics pipeline schematically represented in Supplementary Fig. 1 was utilised to analyse the data. Annotated genes resulting from high quality sequencing reads (Supplementary Fig. 2) were identified in all samples for subsequent downstream analyses. To identify significant gene alterations between unlesioned and MPTP-lesioned groups, a correlation scatter plot of all genes was performed (Fig. 1A) and 143 DE genes were found to be significantly differentially expressed (DE, *P* < 0.05, Fig. 1B, red data-points). From the list of 143 significantly altered genes (Supplementary Table 1), 48 were found to have a greater than 1.2 fold change (FC, Log2), which are comprised of 31 and 17 genes significantly up and down regulated, respectively, in the MPTP-lesioned mice (Fig. 1C). Some of these genes include the down regulation of *Slc6a4* (solute carrier family 6, FC = −3.71), *Mapk13* (mitogen-activated protein kinase 13, FC = −2.82), *Oxt2* (oxytocin, FC = −1.28) as well as upregulation of *Pla2g5* (phospholipase A2, 1.48), *Clic6* (phospholipase A2, FC = 1.54) and *Kcne2* (potassium voltage-gated channel, FC = 5.38) (Fig. 1C).

### Recovery of gene expression in SNpc of MPTP-lesioned mice treated with Cu^II^(atsm)

**Figure 2:**
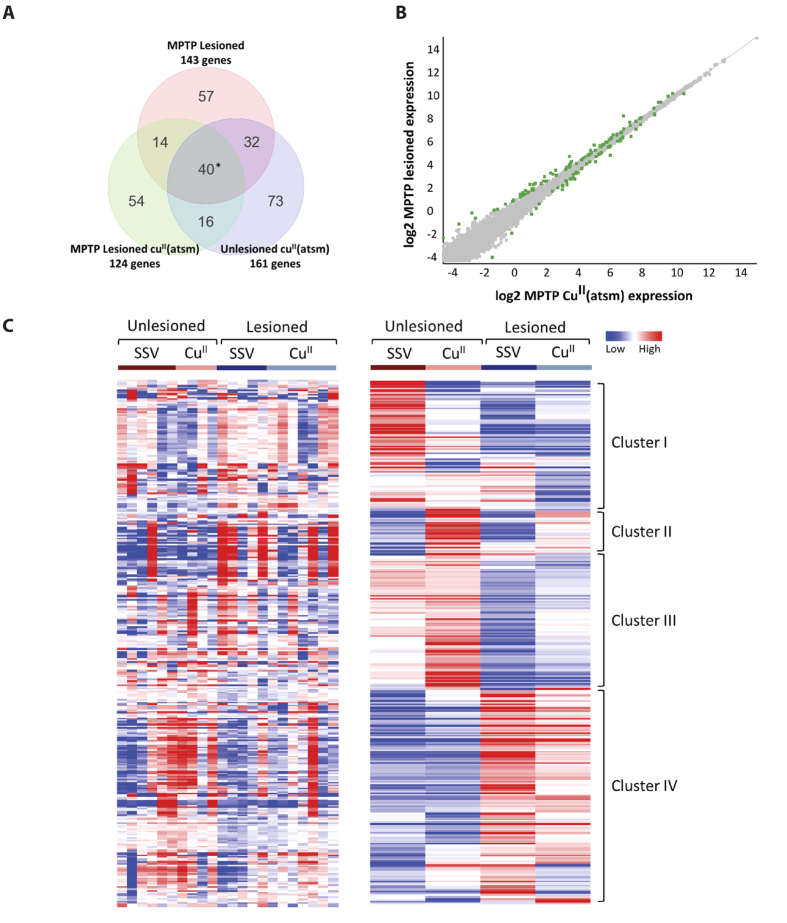
Gene expression distribution in MPTP-lesioned samples treated with Cu^II^(atsm). (**A**) Venn diagram showing the number of common and unique genes between DE genes in (**B,C**). *Genes found to be common in all 3 comparisons with unknown functions (1500015O10Rik, 6330403A02Rik and AI480526) were not considered for qRT-PCR validation. (**B**) Scatter plot highlights 124 significantly DE genes between MPTP Cu^II^(atsm) treated and MPTP lesioned samples (P < 0.05 with Benjamini-Hochberg multiple testing correction) (green data-points). (**C**) Heat map of DE genes expressed across all biological replicates in a pair-wise comparison and the average gene expression across all biological replicates. The profile was found to display common patterns separating genes into 4 clusters. The blue and red gradient represents low- or high- DE genes, respectively. Unlesioned (n = 6), MPTP-lesioned (n = 5), MPTP-lesioned mice treated with Cu^II^(atsm) (n = 7) and unlesioned control treated with Cu^II^(atsm) (n = 4).

MPTP-lesioned mice treated with Cu^II^(atsm), including a group of unlesioned control mice treated with Cu^II^(atsm), also underwent whole transcriptome profiling. Genes DE between MPTP-lesioned mice treated with Cu^II^(atsm) compared to MPTP-lesioned mice (P < 0.05, 124 genes, Fig. 2A-2B, green data-points) were identified. We also compared DE genes between unlesioned mice treated with Cu^II^(atsm) and MPTP lesioned mice (161 genes, P < 0.05, Fig. 2B). Genes with minimal background changes in unlesioned mice treated with Cu^II^(atsm) compared to the unlesioned sham control which are DE upon MPTP lesioning were also analysed. DE genes that changed in unlesioned mice treated upon Cu^II^(atsm) mice compared to the sham controls are consequentially removed through this analysis (Supplementary Table 3). This analysis reveals 40 candidate genes that were specifically recovered upon Cu^II^(atsm) treatment in MPTP-lesioned mice (Fig. 2B and Supplementary Table 2) upon filtering out background effects. Of these 40 genes, three have unknown functions (1500015O10Rik, 6330403A02Rik and AI480526) and were removed for this study leaving a total of 37 genes for further analysis.

Global gene expression patterns of DE genes (P < 0.05, pair-wise comparison) across the treatment groups are displayed in Fig. 2C. The heat map highlights the degree of correlation in gene expression changes across all replicates and their experimental groups (Fig. 2C). The variability within experimental groups may be due to effectiveness of the neurotoxic lesioning as Nissl stains of these mice were observed to display the correct pathology. Nonetheless, there is a distinct pattern of DE genes displayed in Fig. 2C which displays the mean expression values obtained from the replicates in each group.

**Figure 3:**
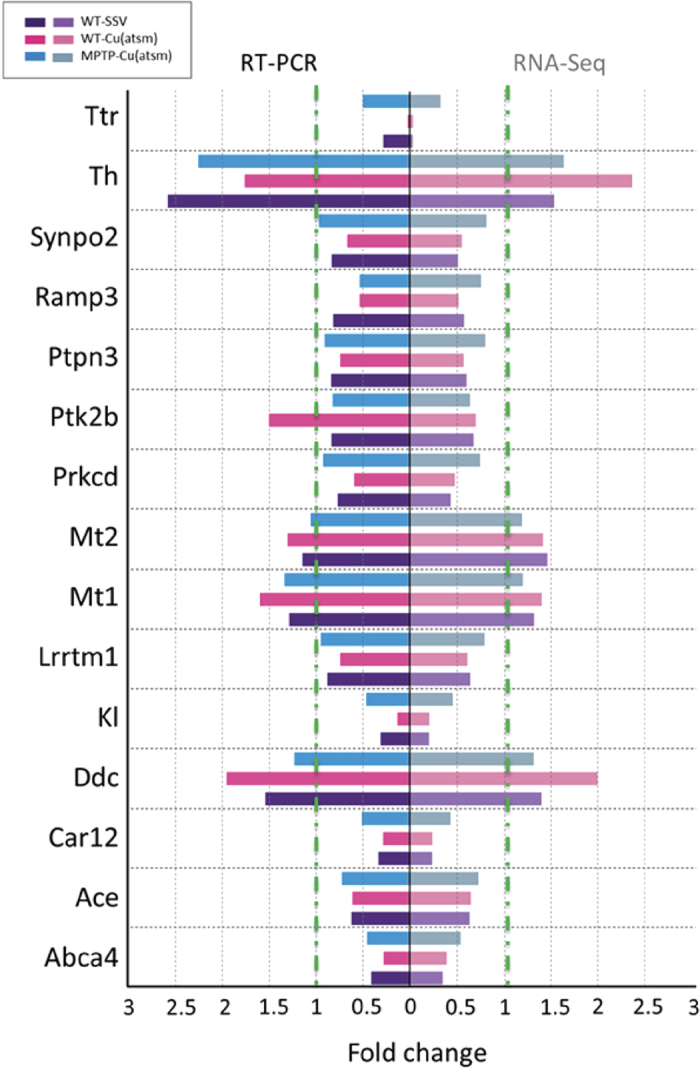
Validation of potential Cu^II^(atsm) gene targets in MPTP-lesioned mice by qRT-PCR. A number of genes were selected to be validated by qRT-PCR to ensure accuracy of the deep sequencing analysis and reproducibility across two methods. Genes identified with a greater than 1.2 fold change (log2) and p-value < 0.05 (cut off at 5% FDR) were chosen. qRT-PCR was performed using TaqMan gene primers specific for the target and normalised to the endogenous control Hprt1. Raw Ct data was uploaded to DataAssist (Applied Biosystems) to calculate fold change using the delta delta Ct (ΔΔCt) method. ANOVA analysis was normalised to MPTP-lesioned mice (green vertical lines). Unlesioned (n = 6), MPTP-lesioned (n = 5), MPTP-lesioned mice treated with Cu^II^(atsm) (n = 7) and unlesioned control treated with Cu^II^(atsm) (n = 4).

Of the 37 genes DE in MPTP-lesioned mice which showed a degree of recovery upon Cu^II^(atsm) treatment, we selected 15 genes for validation using qRT-PCR (Fig. 3). These genes were of particular interest due to evidence in the literature of their association with PD. In most cases, the selected genes display similar fold changes and trend in all treatment groups upon normalisation to MPTP-lesioned mice thus validating the deep sequencing study with qRT-PCR technology (Fig. 3 and Supplementary Table 4). Genes that displayed a notable difference, albeit relatively small, were *Ramp3* and *Mt1* by no more than 0.25 fold change amongst the comparisons (Supplementary Table 4).

### Enrichment analysis of functional and biological related gene groups in MPTP-lesioned mice

The 143 DE genes (False discovery rate, FDR of 5% and P < 0.05) altered in MPTP-lesioned mice and the 37 genes recovered upon Cu^II^(atsm) treatment underwent Gene Ontology (GO) enrichment and pathway analysis which categorised the set of genes into different functional and biological categories (Supplementary Table 5). Using the Database for Annotation, Visualization and Integrated Discovery (DAVID) and MetaCore, GO molecular functional analysis of the selected genes revealed that they were enriched in processes involving binding activity (e.g. calmodulin, receptor and protein) and transporter activity (e.g. transmembrane and substrate-specific; Supplementary Table 6). Biological processes were found to be related to brain and cognitive development, neuroplasticity, regulation and cellular response (Supplementary Table 6). Enrichment analysis was further performed on these genes to determine the cellular localisation of their functions and processes (Supplementary Table 6). Genes were found to be refined in cellular components such as the cell periphery, plasma membrane, extracellular region, cell projection, synapse junctions and vesicle secretion/formation (Supplementary Table 6). The analyses highlighted key biological and neurobiological processes, functions and pathways which were then used to further analyse the altered biological pathways in MPTP-lesioned mice.

### Pathway analysis of DE genes upon MPTP lesioning and Cu^II^(atsm) treatment

**Figure 4:**
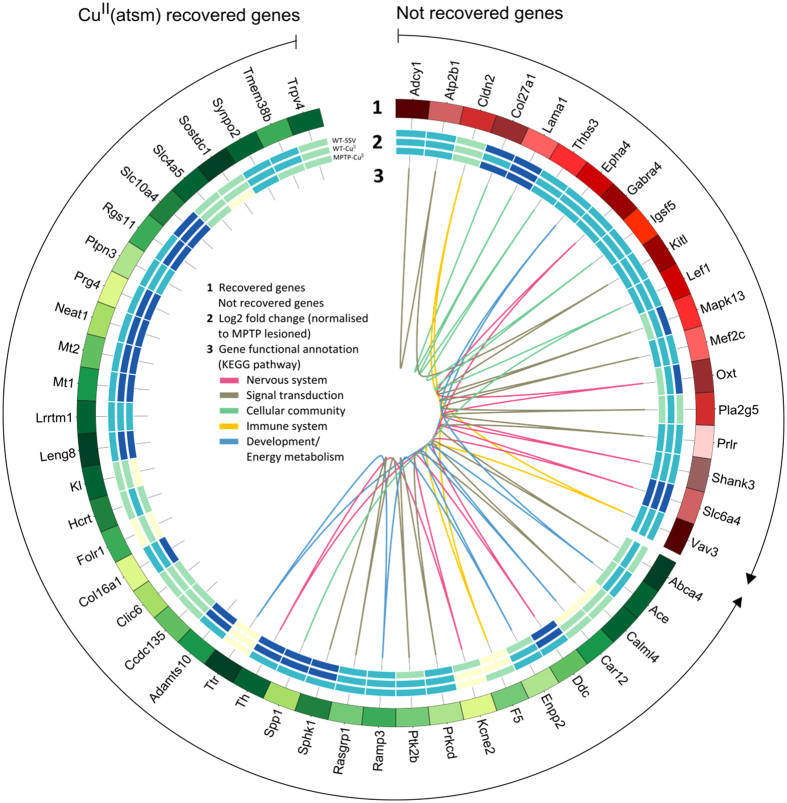
Circos diagram depicting Cu^II^(atsm) and MPTP-lesioned transcriptome data. Track 1: Genes related to Cu^II^(atsm) recovered (green shades) and not recovered genes (red shades). The two arrows represent the start and end point of genes that are in Cu^II^(atsm) recovered and not recovered data. Track 2: Heatmap expression of WT-SSV, WT-Cu^II^(atsm) and MPTP-Cu^II^(atsm). Expressions are represented in log2 fold change, where values are normalised to MPTP lesioned. The degree of low to high expression is denoted by a range of colour starting from light to dark blue. Track 3: Biological pathways enriched among Cu^II^(atsm) and MPTP-lesioned genes. More specifically, cellular community encompasses tight junctions, gap junctions, adheren junctions and focal adhesion molecules.

The common regulatory pathways affected upon MPTP lesioning and those recovered upon Cu^II^(atsm) treatment can be visualised in the circosdiagram in Fig. 4, which visually represents expression changes across treatments. The circosdiagram illustrates enrichment of genes in the nervous system, immune system, cellular community, development/energy metabolism and signal transduction pathways (Fig. 4). Genes classified in the same biological pathway across both databases demonstrates high validity and include *Adcy1* (adenylatecyclase 1), *Mapk13* (p38 delta MAP kinase), *Prkcd* (protein kinase C, delta) and *Ptk2b* (PTK2 protein tyrosine kinase 2 beta). To understand the relationship of these genes, *Adcy1*, *Mapk13, Prkcd* and *Ptk2b* were used to identify direct and indirect relationships with the other 143 DE genes identified in MPTP-lesioned mice.

**Figure 5:**
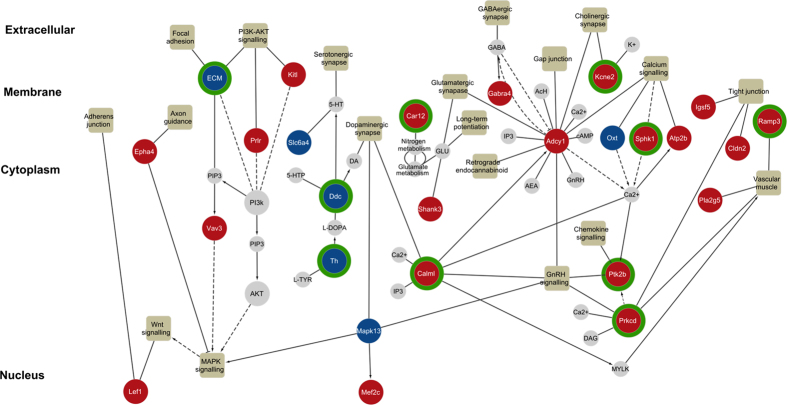
Pathway map detailing gene expression changes in the identified biological pathways via enrichment analysis. Extracellular matrix (ECM) genes include Col27a1, Lama1, Thbs3 and Spp1. Compounds and genes are represented by circles (nodes). Genes identified in the data are coloured according to the degree of fold change; up-regulated (red) and down-regulated (blue). Genes displaying a green border recovered upon Cu^II^(atsm) treatment. Compounds and genes which associate with gene interests are coloured as grey and connected with black solid edges. Pathways are represented by squares. Solid and dash arrows indicate direct and indirect reaction respectively.

To identify the key biological pathways in which the DE genes are representing, a pathway map (Fig. 5) along with the genes of interests and signalling pathways were compiled to visualise the implication of gene expression changes after MPTP lesioning and Cu^II^(atsm) treatment. The map contains 19 pathways, including enrichment pathways and 29 DE genes. Observations from the map uncovered that the DE genes were typically found at the extracellular and membrane regions that affected intrinsic signalling events. The combined approach allowed the identification of DE genes that have been directly or indirectly implicated in PD in this mice model including those targeted by Cu^II^(atsm) (Fig. 5). Within these enriched pathways, Cu^II^(atsm) was found to target key regulatory genes such as *Prkcd*, *Ptk2b*, *Th*, *Ramp3* and *Calml* (Fig. 5).

## Discussion

Current therapeutic strategies for PD largely involve dopamine replacement with L-Dopa (Levodopa) or dopamine receptor agonists (Cabasar and Ropinirole) which provide symptomatic relief. In the search for new neuroprotective drugs, various compounds have been shown to rescue PD phenotypes in MPTP-induced mice models^14^,^15^. For example, M30, an iron chelalor, has been found to restore the activity of nigrostriatal dopamine neurones in MPTP mice models through the ability to activate the hypoxia-inducible factor (HIF)^15^,^16^. In our previous study, the beneficial effects of the potential neuroprotective drug Cu^II^(atsm) seen in MPTP-lesioned mice was attributed to its peroxynitrite scavenging abilities thus decreasing protein nitration and retaining neuronal integrity. However, it is likely that Cu^II^(atsm) also mediates other biological activities thus we performed whole transcriptome gene expression sequencing. Unlike microarray^8^,^17^ and qRT-PCR methodology^18^,^11^ whole transcriptome sequencing performed in this study has allowed an in-depth global assessment of all genes Cu^II^(atsm) may target in the SNpc. This study is also the first unbiased whole transcriptome analysis performed on MPTP-lesioned mice which covered 25, 902 genes. This study also addresses whether the MPTP lesioning represents similar aspects of PD in humans at the transcriptional level, and by doing so determine to what degree MPTP toxicity can predict translational targets in PD research. The deregulated genes we identify in the network collectively point towards dopamine synthesis impairment and perturbed synaptic neurotransmission, consistent with their involvement in PD.

In this study, the level of *Th*, *Ddc and Slc6a4* gene expression decreased which supports the destruction of dopaminergic neurons and loss of dopamine in injured neurons observed in the MPTP model system^2^,^19^,^20^. A decrease in *Th* expression has also been detected in microarray studies performed on MPTP-treated SNpc tissue^17^,^21^ and validated by qRT-PCR in both SNpc and striatum tissue from MPTP lesioned mice across 3–24 hr timepoints^11^. Low levels of dopamine contribute to the clinical manifestations of muscle stiffness and slowness of movement in PD as these neurotransmitters control coordination and smooth movement. Deregulated *Ddc* not only perturbs the normal biosynthesis of dopamine but also serotonin regulated by the *Slc6a4* (SERT) gene. *Slc6a4* is involved in serotonin uptake and synaptic vesicle trafficking making this transporter protein a potential neuroprotective target. For example, Dextromethorphan, a SERT inhibitor, was found to be neuroprotective for dopaminergic cells in the MPTP-diethyldithiocarbamate (DDC) model of Parkinsonism^22^. Furthermore, serotonin and dopamine levels were found to increase upon treatment of MPTP-lesioned mice with M30^23^. Compared to other studies, other members of the solute carrier family were found to be deregulated such as *Slc6a11*^8^,^21^. These differences may be attributed to the different MPTP doses and tissue sections used amongst studies^8^,^17^,^21^. Nonetheless, the alterations of these key PD associated genes within the SNpc reveal that the site of MPTP toxicity may be within the presynaptic terminal of the dopaminergic neurones which subsequently causes the loss of neurotransmission and the phenotypic observations seen in the model. These observations support that the MPTP model is able to recapitulate major hallmarks of the human disease. Furthermore, this exposes possibilities to design neuroprotective compounds that specifically modify gene expression.

It has been reported that the MPTP neurotoxin also causes an increase in nitrosative stress^24^ however, we did not detect gene expression changes directly associated with nitrostative stress. Rather we detected changes in genes associated with nitrogen-glutamate metabolism (*Shank3*; SH3 and multiple ankyrin repeat domains 3 and *Car12*; carbonic anyhydrase 12) and calcium signalling (*Adcy1, Oxt, Sphk*; Sphingosine kinase 1 and *Atp2b*) as identified through KEGG pathway analysis. Overall, calcium homeostasis is critical in neuronal cells and regulates many functions such as cell survival and promoting neuronal function and communication^25^. Calcium also modulates both reactive oxygen species (ROS) and nitric oxide (NO) generation, including clearance in the mitochondria and cytosol. The roles of these genes indirectly and directly associated with calcium signalling within the MPTP model need to be further validated to understand whether the alteration causes an upstream or downstream effect in calcium dysregulation or neuronal stress through ROS/NO.

Cellular communication through tight junctions, gap junctions, adherens junctions and focal adhesion molecules are essential in the nervous system for the transmission of nerve signals^26^,^27^. In this study, increased gene expression of *Igsf5* (immunoglobulin superfamily, member) and *Cldn2* (claudin 2) were perceived to maintain cell polarity upon the loss of neurons after MPTP treatment. However, genes involved with focal adhesion and the extra cellular matrix (ECM), *Col27a1* (collagen, type XXV^II^, alpha 1), *Thbs3* (thrombospondin 3), *Spp1* (secreted phosphoprotein 1) and *Lama1* (laminin, alpha 1) were downregulated, suggesting the existence of damaged neurons. Prior reports from Chang *et al.* highlighted that matrix metalloproteinase-3 (MMP-3), a proteinase that degrades ECM components, is released following damage to dopaminergic neurons and compromises the BBB after MPTP treatment^28^. Gap and adheren junctions are critical in cell-to-cell communication through maintaining the homeostasis of the epithelium layer of the BBB^29^ and communicating changes between the brain and blood stream^30^. Thus, future studies should involve whole transcriptome sequencing on other sections of the brain in MPTP lesioned mice such as the striatum.

A major hallmark of PD is the malfunction of presynaptic signalling, however what occurs post-synaptically and intracellularly is less defined. It has been shown that chemokine signalling increases in MPTP-induced mice and activation of this pathway leads to cell adhesion, cell survival, neuronal polarity and synaptic transmission^31^. The attributes of chemokine signalling were reflected in the enrichment analysis of GO biological processes and pathways identified in this study. Genes involved in intracellular signal transduction relating to cell stress, cell death or survival^32^,^33^ include *Mapk13*, *Mef2c* (Myocyte Enhancer Factor 2c) and *Lef1* (lymphoid enhancer binding factor 1). PI3k-Akt signaling pathway is well-known for cell growth and survival. Activation of genes for *Kitl* (kit ligand), *Prlr* (prolactin receptor) and *Vav3* (vav 3 oncogene) may mediate signals for reduction of apoptosis to avoid a major cascade of neuronal death in response to MPTP neurotoxicity. Other chemokine signalling genes identified were *Prkcd* and *Pla2g5.* Although these genes are enriched in the GO enrichment pathway related to vascular muscle, these genes also mediate membrane phospholipid synthesis. Studies have shown that phospholipase A2 show less activity in the SNpc of PD patients which may result in the inability to turnover phospholipids at the neuronal membrane upon oxidative stress^34^. The loss of phospholipase A2 activity may illicit a positive feedback loop to upregulate the transcription of the phospholipase A2 family of enzymes such as *Pla2g5* as seen in MPTP-lesioned mice. Group 6 of the phospholipase A2 (also known as *Park14*) family has been well-characterised to be associated with PD in which single-nucleotide polymorphisms have been identified in PD patients^35^. Conversely, Group V of the phospholipase A2 family have been implicated in inflammation^36^. These genes identified in post-receptor signalling show activation of cell survival and growth with compensational activation of cell stress and death. This may explain the lack of therapeutic drugs aimed at targeting post-receptor signalling molecules.

The *Ttr* (transthyretin) gene was found to be one of the most upregulated (FC = 4.9, P < 0.01) genes in SNpc of MPTP-lesioned mice. Transthyretin has been shown to be indirectly associated with PD by its presence in serum and cerebrospinal fluid in Lewy body disorders^37^. Conversely, the most downregulated gene was *Fat2* (FAT Atypical Cadherin 2, FC = −4.1, P = 0.00), a cadherin protein found restricted to the nervous system in the brain, which initial studies have shown its involvement in the development of neurons^38^. In addition, a number of homeobox genes (*Hoxc4, Hoxb3os, Hoxb3* and *Hoxb5, FC* > *1.2*, P < *0.05*) were also found to be significantly downregulated in MPTP mice. These represent a highly conserved family of transcription factors involved in neurogenesis such as neuronal differentiation and neuronal connectivity (reviewed in^39^). Other homeobox genes associated with neurogenesis such as *Pitx3* (paired-like homeodomain transcription factor 3) has been shown to in involved in the development of dopamine neurons of the SNpc^40^. In this study, *Pitx2* (paired-like homeodomain transcription factor 2) was found to show a modest but significant fold change (FC = 0.9, P < 0.05). The role of *Hoxc4, Hoxb3os, Hoxb3* and *Hoxb5* should be investigated further in the MPTP mice which may further support the possibility of regenerating neurones in the SNpc through new therapies^41^.

This is the first report revealing possible neuroprotective mechanisms of Cu^II^(atsm) through understanding changes in gene regulation. Some of the restored genes include *Ddc*, *Th*, *Calml*, *Kcne2*, *Sphk1*, *Ptk2b*, *Prkcd* and *Ramp3* which are associated with dopamine synthesis, synaptic neurotransmission and calcium signalling all required for coordination and smooth movement^2^,^19^,^20^,^42^. This may explain the significant improvement of motor function in MPTP lesioned mice upon treatment with Cu^II^(atsm) where an increase of TH and dopamine was confirmed by stereological counting of Nissl stained SNpc neurones and Western blotting^2^. Other restored genes were carbonic anhydrases such as *Car12* which can also modulate neuronal excitability through mediating levels of H^+^ ions. H^+^ controls pH within and outside of neurones which is fundamental to neuronal development and synaptic plasticity^43^. Furthermore, the regulation of H^+^ in the brain requires the conductance of ionic channels and gap junctions to function which associated genes were found to be DE in MPTP-lesioned mice (*Kcne2*, *Slc10a4*, *Col27a1*, *Thbs3*, *Lama 1 and Spp1)* and the majority restored upon Cu^II^(atsm). This suggests that Cu^II^(atsm) may not only promote the survival of dopaminergic neurones but has downstream effects to restore excitability within tissue by promoting the flow of ions through junctions of adjoining cells. Furthermore, Cu^II^(atsm) treatment restored metallothionein 1 and 2 (*Mt1* and *Mt2*) to unlesioned levels by up regulating their expression supporting reported neuroprotective effects of metallothionein (MT) observed in the literature^44^,^45^,^46^. Overexpression of metallothionein in the brain of MT-transgenic mice inhibited the nitration of α-synuclein and peroxynitrite-induced cell death^46^. *Ttr* was also restored in MPTP-lesioned mice treated with Cu^II^(atsm). Using qRT-PCR, changes in *Ttr* expression were observed across various sections of the brain in adult unlesioned C57BL mice treated with M30^18^. The detection of *Ttr* before and after treatment may support its use as a potential biomarker for monitoring disease progression^47^.

The current transcriptomic data provides detailed signalling insights into potential drug targets where disease-modifying drugs can aim to restore dopamine metabolism, promote neurogenesis and re-establish normal homeostatic balance in the synaptic space and within neurons. Here, Cu^II^(atsm) may serve as a promising neuroprotective drug to prevent the destruction of dopaminergic neurons and impairment of dopamine synthesis in PD. The validation of gene expression levels highly associated with PD such as *Th, Ddc* and solute carrier family genes suggests that the other differentially expressed genes identified in this study should be investigated further to uncover new insights into PD pathology and new potential treatments.

## Materials

### Study design

All studies and analysis were conducted in a blinded fashion to remove any bias associated with analysis. MPTP lesioning of C57BL/6 mice was performed as previously published^2^. Briefly, four doses of MPTP (Sigma-Aldrich) were injected intraperitoneally into C57BL/6 mice at a dose of 10 mg/kg at 2-h intervals, the total dose per mice being 40 mg/kg. A cohort of mice were sham lesioned with saline, and then orally gavaged with SSV for the duration of the trial of 19 d. Another group of unlesioned and MPTP-lesioned mice was treated with 30 mg/kg Cu^II^(atsm) via oral gavage for 21 d. Pole test was performed after 19 d of treatment which was used to assess specific motor impairments related to basal ganglion pathology as described in our previous study^2^. Animals were killed by an overdose of sodium pentobarbitone (100 mg/kg Lethobarb; Jurox), and perfused via the heart with cold 0.1 M PBS (Sigma-Aldrich), pH 7.4. The left brain hemisphere was dissected into regions and the SNpc was treated in RNAlater (Life Technologies) to preserve RNA for isolation. The study involved unlesioned (*n* = 6), MPTP-lesioned (*n* = 5) and MPTP-lesioned mice treated with Cu^II^(atsm) (n = 7) including a unlesioned control treated with Cu^II^(atsm) (n = 4). All methods conformed to the Australian National Health and Medical Research Council published code of practice for animal research, and experimentation was approved by a University of Melbourne Animal Ethics Committee. Sprague Dawley rats and C57BL/6 mice were purchased from Monash Animal Services.

### RNA isolation, whole transcriptome library preparation and deep sequencing

Total RNA was isolated from SNpc section of all mice in the study using the miRNeasy kit (QIAGEN) according to manufacturer’s instructions. RNA integrity was assessed using the RNA Nano 6000 kit and run on the Agilent 2100 Bioanalyser (Agilent Technologies). mRNA was further isolated from total RNA using the NEBNext Poly(A) mRNA magnetic isolation module (New England BioLabs) according to manufacturer’s instructions and the yield and size distribution of mRNA was analysed using a RNA Nano 6000 chip. The mRNA samples were fragmented in preparation for cDNA synthesis and library construction using the NEBNext mRNA Library Prep Master Mix Set (New England BioLabs), whereby the manufacturer’s instructions were followed including ligating each sample with a unique index primer using the NEBNext Multiplex Oligos for Illumina (New England BioLabs). Library quality was assessed on an Agilent 2100 Bioanalyser using a DNA 1000 chip prior to sequencing on the Illumnia Hiseq 2500. Sequencing was performed at the Centre for Translational Pathology, Department of Pathology, University of Melbourne. Raw files can be downloaded at ENA, primary accession # PRJEB6957.

### Reads quality control and assembly

A basic differential expression analysis pipeline (https://www.vlsci.org.au/pipelines_and_protocols) developed by Life Sciences Computation Centre at the University of Melbourne was employed to process and align sequence reads generated from the libraries. The pipeline was based around Tuxedo protocol and R bioconductor packages. Briefly, sequence read quality was initially assessed in Illumina BaseSpace and parsed to FastQC for further evaluation. Only those reads with at least 80% of the base calls above Q30 were retained. The 3′ adapter sequence was removed from all reads using the suggested parameters in Trimmomatic^48^. The following parameters were used: ILLUMINACLIP:TruSeq3-SE:2:30:10 LEADING:3 TRAILING:3 SLIDINGWINDOW:4:15 MINLEN:36. Quality-filtered single-end sequence reads were then aligned to the Ensembl GRCm38 *Mus musculus* reference genome using TopHat version 2.0.8^49^, which incorporates the Bowtie version 2.1.0^50^ algorithm to perform alignment and also SAMtools version 0.1.19^51^ for alignment output formatting. Alignment parameters were set to the protocol described in^52^. Subsequently, alignment statistics can be generated using RNA-SeQC for quality control of these aligned reads.

### Differentially expressed gene analysis

The R package edgeR version 3.8.5^52^ and HTSeq version 0.6.1^53^ were used respectively to perform gene count and differentially gene expression analysis. Aligned reads that mapped to the mouse gene annotation from Ensembl were identified using HTSeq with the default *htseq-count* functionality. Gene quantification in text format was generated via HTSeq. In order to measure gene expression differences between case and control samples, edgeR use model count data by a negative binomial distribution to assess the quantitated reads. Read counts were normalised using weighted trimmed mean of M-values. The normalised reads within edgeR were attained using the *calcNormFactors* function. The normalised expression values were displayed as counts per million (cpm) and read counts were loaded into Seqmonk version 0.27.0 for visualisation (www.bioinformatics.babraham.ac.uk/projects/seqmonk). To determine fold change differences, the log of ratio of expression levels for each gene between conditions being tested was computed. Significantly DE genes were identified as those with a *P* value of <0.05 with Benjamini-Hochberg multiple testing correction at 5% FDR and a fold change to be a minimum of 1.2 fold change (log2). We consider this a reliable criteria which identifies significant genes of interest with notable differences in fold change which can be validated and reproduced by qRT-PCR using other biological replicates.

### Functional and pathway enrichment analysis

To reveal GO biological processes, molecular functions and localisations between the altered genes in MPTP-lesioned mice treated with and without Cu^II^(atsm) treatment, DE genes (P < 0.05, FDR 5%), were queried using The Database for Annotation, Visualisation and Integrated Discovery (DAVID 6.7)^54^ and GeneGoMetaCore^®^ software (GeneGo, Inc., Encinitas, CA, USA). The pathways were then classified using the Kyoto Encyclopedia of Genes and Genomes database^55^ by their functional hierarchy schema according to major functions. Different pathways in the network were analysed using Merge Network and cytoHubba plugin to assess gene interactions using default parameters in Cytoscape version 3.1.0^56^. The pathways and their corresponding genes were visualised using Circos version 0.64^57^ to illustrate the impact of the genes of interest in this study.

### Statistical analysis

Preliminary quality control of sequencing reads was determined by the Phred scoring system. A Phred score of a base is: 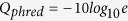, where 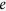 is the error probability. Differential expression across treatment groups was performed with edgeR and visualised on SeqMonk with a corrected p-value, factoring in multiple comparison adjustment for multiple testing with Benjamini-Hochberg correction to control for FDR. FDR and p-value cutoff is set at below 0.05. Here, a negative binomial distribution was used to perform statistical testing. The library counts are modeled as follows: 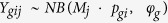
58, where 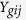 is the counts for transcript *g* in group *i* and replicate *j*; 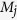 is the library size of replicate *j*; 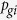 the fraction of reads for transcript *g* and group *i*; 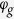 is the over dispersion for transcript *g* representing the variability. Pearson correlation coefficient was used to examine the relationship between different samples and the distribution was graphed in a scatter plot. To assess gene function and pathway, enrichment analysis was performed using the default parameters in DAVID and GeneGoMetaCore^®^ through implementing Fisher’s exact test and adjusted for multiple testing using Benjamini-Hochberg FDR analysis to obtain significant results (P Value < 0.05, FDR < 0.05)^54^. To test the statistical significance of genes intersecting within a pathway/network, hypergeometric distribution model was used. The p-value was modeled as follows: 
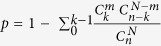
59, in which *N* is the total number of genes in the pathways; *n* is the number of gene interests; *m* is the number of gene samples in one pathway; *k* is the frequency of genes overlapped in all pathways.

### qRT-PCR validation

For qRT-PCR, 1.5 μg of RNA was converted to cDNA (TaqMan cDNA Reverse Transcription Kit, Applied Biosystems, Australia) according to the manufacturers’ protocol. qRT-PCR (TaqMan Fast Advanced Master Mix, Applied Biosystems) was performed using individual gene assays (TaqMan gene assays, 20x, Applied Biosystems) and run on the VIIA™ 7 Real-Time PCR System (Life Technologies). Each biological replicate (unlesioned (*n* = 6), MPTP-lesioned (*n* = 5), MPTP-lesioned Cu^II^(atsm) (n = 7) and unlesioned Cu^II^(atsm) (n = 4)) was run in triplicate to monitor technical variability and quality control. For data normalisation across samples, Hprt1 was used as an endogenous control gene. Normalisation of Ct values of each gene and determination of fold differences in gene expression (normalised to MPTP-leisoned mice) was calculated by the 2^−∆∆Ct^ method. Data assist (Applied Biosystems) was used to analyse the data.

## Additional Information

**How to cite this article**: Cheng, L. *et al.* Gene dysregulation is restored in the Parkinson’s disease MPTP neurotoxic mice model upon treatment of the therapeutic drug Cu^II^(atsm). *Sci. Rep.* **6**, 22398; doi: 10.1038/srep22398 (2016).

## Supplementary Material

Supplementary Information
