# Supplementary material for: Gene dysregulation is restored in the Parkinson’s disease MPTP neurotoxic mice model upon treatment of the therapeutic drug CuII(atsm)
Source: Sci Rep. 2016 Mar 1;6:22398. doi: 10.1038/srep22398 (PMC4772163; doi:10.1038/srep22398)
Supplement: Supplementary Information [file srep22398-s1.pdf]

<u>Supplementary Figures and Tables</u>

Gene dysregulation is restored in the Parkinson's disease MPTP neurotoxic mice model upon treatment of the therapeutic drug Cu^{II}(atsm).

Lesley Cheng[1,3,5*], Camelia Y.J. Quek[1,3*], Lin W. Hung[3,4], Robyn A. Sharples[1,3], Nicki A. Sherratt[2,3],

Kevin J. Barnham[2,3,4], and Andrew F. Hill[1,3,5§]

Supplementary Figure 1:

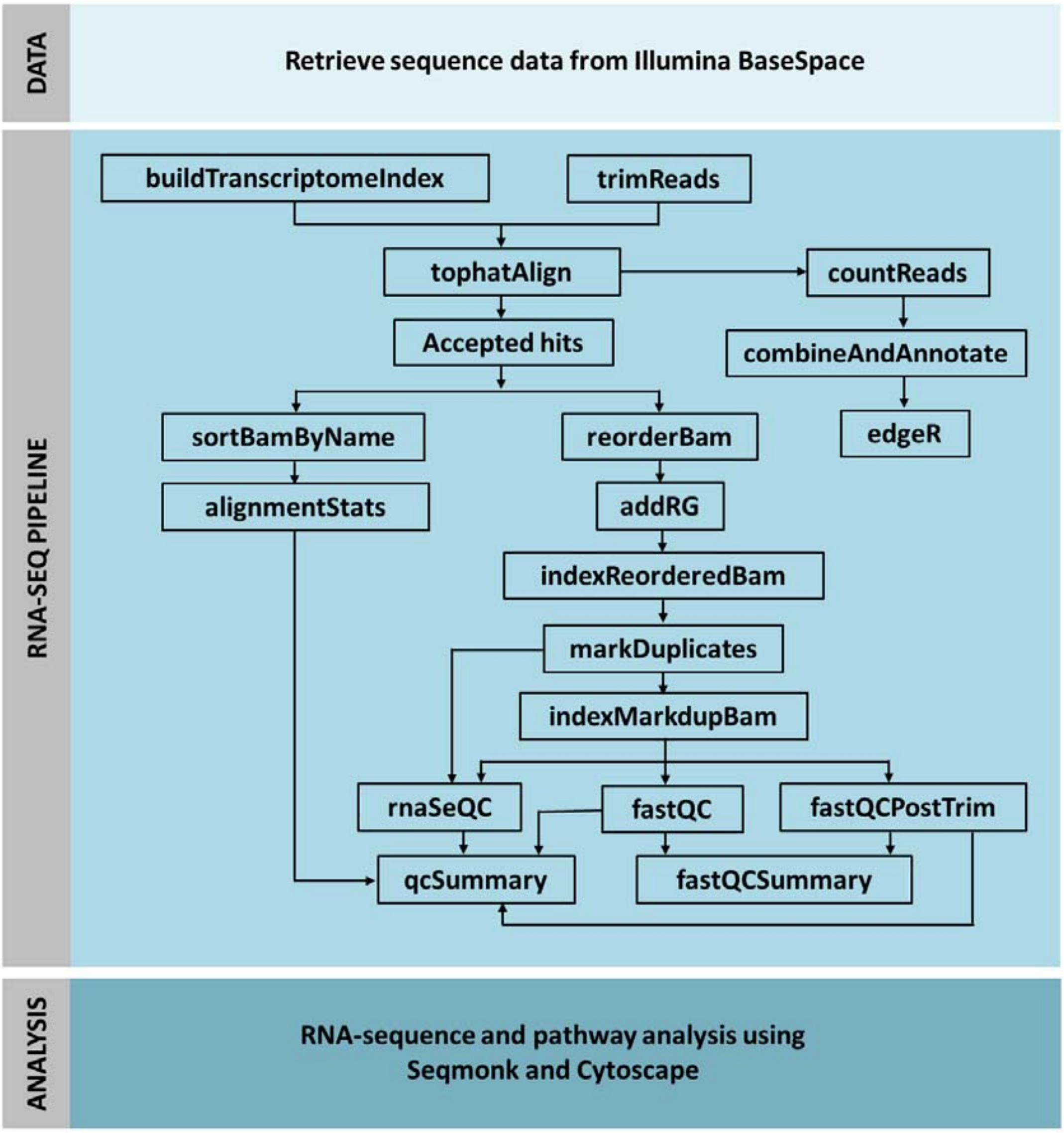

SF1: Workflow of RNAseq data analysis. Raw sequences from each sample are retrieved from Illumina BaseSpace followed by bioinformatics analysis as shown.

Supplementary Figure 2:

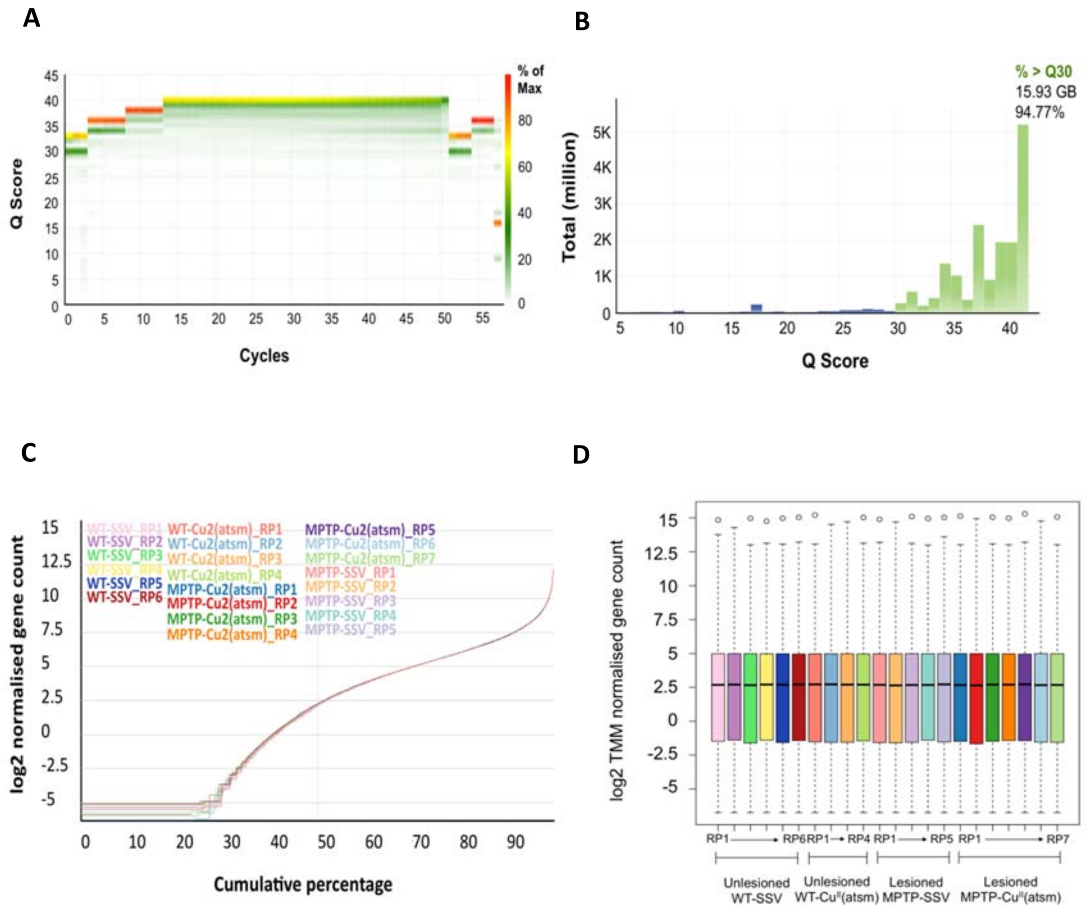

SF2: Quality and global normalisation of sequencing data. (A) A heatmap of the Quality (Q) Score displays an overview of Q Score over the cycles. The Q score is cumulative for the current cycle and previous cycles and an average of Q40 is achieved as the cycle progresses. **(B)** The Q Score distribution plot shows the number of bases by Q Score for all reads across all cycles, where Q score is based on the Phred Scale ranging from Q10 to Q40. Most of the sequencing data (~94.77%) has achieved more than Q30. **(C)** A cumulative distribution plot to assess the normalisation of all samples. The x-axis shows an order of genes distributing from lowest to highest percentile. The y-axis shows the normalised logarithm base 2 (log2) value that the genes have in those samples. The similar profiles obtained after normalisation shows appropriate methods were used. **(D)** To examine the global gene expression profiles, read counts of individual genes were quantitated, normalised and log transformed. Similar distribution profiles of normalised gene counts were observed across the whole distribution for all samples on the plot. The profiles indicated that samples were appropriately normalised. Unlesioned (n = 6), MPTP-lesioned (n = 5), MPTP-lesioned mice treated with Cu^{II}(atsm) (n = 7) and unlesioned control treated with Cu^{II}(atsm) (n = 4).

Supplementary Figure 3:

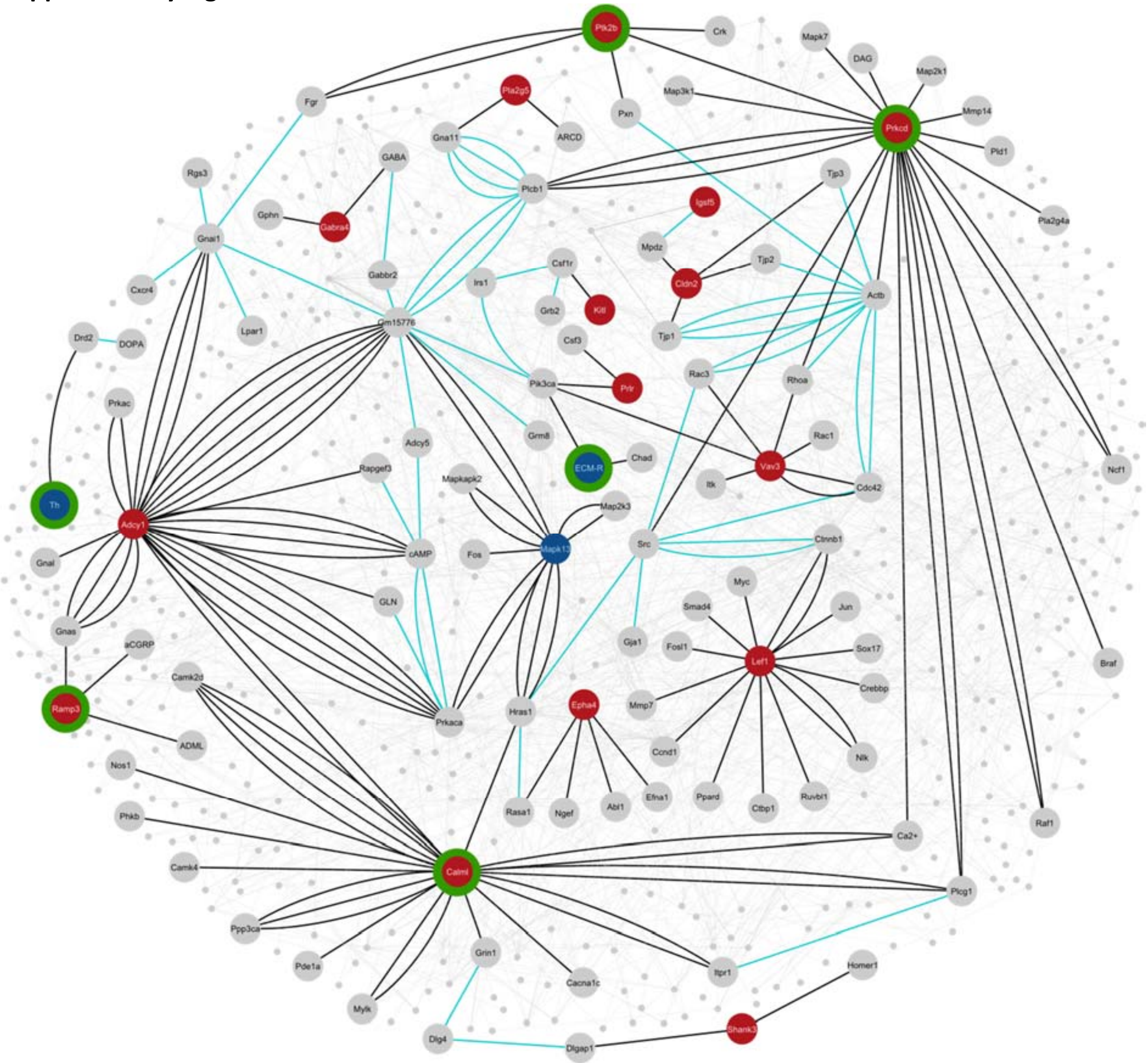

SF3: Interactive PDF of Figure 5A. Pathways of differentially expressed genes involved in MPTP-induced model of Parkinson's disease. An interactive map highlighting the interaction of genes of interests centring Adcy1, Mapk13, Prkcd and Ptk2b among multiple mapped pathways which can be virtuallyviewed online. Node denotes genes or compounds, while edge denotes the link between the two nodes. Gene interests are connected by direct (bolded solid black edges) or intermediate interaction (solid blue edges) involved in the same or different pathways. Genes identified in the data are coloured according to the degree of fold change; up-regulated (red), down-regulated (blue) and no expression change (grey).

Supplementary Tables

Supplementary Table 1: 143 differentially expressed genes ($p<0.05$, FDR < 5%)

Description of the data: Significant differentially expressed genes were identified as those with a *P* value of < 0.05 with Benjamini-Hochberg multiple testing correction at 5% false-discovery rate (FDR). The comparison expression plot revealed 143 genes that were differentially expressed.

Supplementary Table 2: 37 genes DE in MPTP lesioned mice which showed a degree of recovery upon Cu^{II}(atsm) treatment

Description of the data: MPTP lesioned mice treated with Cu^{II}(atsm), including a group of unlesioned control mice treated with Cu^{II}(atsm), also underwent whole transcriptome profiling. Data analysis was performed to filter for genes that recovered upon Cu^{II}(atsm) treatment.

Supplementary Table 3: DE genes in unlesioned mice treated with Cu(atsm) compared to unleisoned sham controls

Description of the data: Analysis of genes showing differentially expressed genes upon comparing unlesioned mice treated with Cu^{II}(atsm) compared to unlesioned SSV mice.

Supplementary Table 4: Validation of fold expression changes between MPTP lesioned mice and upon treatment with Cu^{II}(atsm) by qRT-PCR

Description of the data: Fold change calculated from the validation of Cu^{II}(atsm) targets in MPTP lesioned mice by qRT-PCR. For data normalisation across samples, HPRT was used as an endogenous control gene. Normalisation of Ct values of each gene and determination of fold differences in gene expression (normalised to MPTP leisoned mice) was calculated by the $2^{-\Delta\Delta Ct}$ method. Data assist (Applied Biosystems) was used to analyse the data.

Supplementary Table 5: Functional categories

Description of the data: Gene Ontology (GO) enrichment and pathway analysis for the differentially expressed genes in MPTP-lesioned mice.

Supplementary Table 6: Enrichment Analysis

Description of the data: Database for Annotation, Visualization and Integrated Discovery (DAVID) and MetaCore, GO molecular functional analysis of all differentially expressed genes.

Supplementary Table 1: 143 differentially expressed genes (p<0.05, FDR < 5%)

Gene	Chromosome	Start	End	Strand	Diff p-value	Ensembl ID	Description	Log2 Fold-change	Gene cluster
1500015O10Rik	1	43730602	43742578	+	0.0000000058	ENSMUSG00000026051	RIKEN cDNA 1500015O10 gene [Source:MGI Symbol;Acc:MGI:1926146]	3.1024804	2
4930547N16Rik	10	88091432	88146941	-	0.0000168365	ENSMUSG00000035365	RIKEN cDNA 4930547N16 gene [Source:MGI Symbol;Acc:MGI:1922567]	0.8969573	2
6330403A02Rik	1	180432387	180483504	-	0.0158841310	ENSMUSG00000053963	RIKEN cDNA 6330403A02 gene [Source:MGI Symbol;Acc:MGI:2138735]	0.511204	2
6330527O06Rik	2	136052239	136069917	+	0.0111422550	ENSMUSG00000027270	RIKEN cDNA 6330527O06 gene [Source:MGI Symbol;Acc:MGI:1923411]	-0.690673	1
Abca4	3	122044443	122180061	+	0.0002153724	ENSMUSG00000028125	ATP-binding cassette, sub-family A (ABC1), member 4 [Source:MGI Symbol;Acc:MGI:109424]	1.4490194	2
Abhd12b	12	70154142	70183347	+	0.0009762756	ENSMUSG00000090121	abhydrolase domain containing 12B [Source:MGI Symbol;Acc:MGI:2685650]	0.8073483	2
AC149868.1	7	45176349	45179597	-	0.0218575400	ENSMUSG00000095276	uncharacterized protein LOC210541 precursor [Source:RefSeq peptide;Acc:NP_001182184]	-2.74934475	1
AC151712.4	Y	90784738	90816464	+	0.0001523704	ENSMUSG00000096768	erythroid differentiation regulator 1 [Source:RefSeq peptide;Acc:NP_579940]	-0.5881615	1
Ace	11	105967945	105989964	+	0.0283734850	ENSMUSG00000020681	angiotensin I converting enzyme (peptidyl-dipeptidase A) 1 [Source:MGI Symbol;Acc:MGI:87874]	0.5935577	2
Acrbp	6	125049689	125063267	+	0.0036505444	ENSMUSG00000072770	proacrosin binding protein [Source:MGI Symbol;Acc:MGI:1859515]	-0.9281974	1
Adamts10	17	33524204	33553782	+	0.0000014718	ENSMUSG00000024299	a disintegrin-like and metallopeptidase (reprolysin type) with thrombospondin type 1 motif, 10 [Source:MGI Symbol;Acc:MGI:2449112]	-0.7551333	1
Adcy1	11	7063489	7178506	+	0.0000210313	ENSMUSG00000020431	adenylate cyclase 1 [Source:MGI Symbol;Acc:MGI:99677]	0.6738905	2
AI480526	5	123133723	123141666	-	0.0073642720	ENSMUSG00000090086	expressed sequence AI480526 [Source:MGI Symbol;Acc:MGI:2140910]	-0.6191359	1
Aqp1	6	55336432	55348555	+	0.0000703560	ENSMUSG00000004655	aquaporin 1 [Source:MGI Symbol;Acc:MGI:103201]	3.2030673	2
Arrdc1	2	24925352	24935252	-	0.0198427800	ENSMUSG00000026972	arrestin domain containing 1 [Source:MGI Symbol;Acc:MGI:2446136]	-0.56512	1
Atp2b1	10	98915152	99026143	+	0.0120894560	ENSMUSG00000019943	ATPase, Ca++ transporting, plasma membrane 1 [Source:MGI Symbol;Acc:MGI:104653]	0.5604734	2
Calml4	9	62858104	62875918	+	0.0264056100	ENSMUSG00000032246	calmodulin-like 4 [Source:MGI Symbol;Acc:MGI:1922850]	1.710185732	2
Car12	9	66713686	66766845	+	0.0000000000	ENSMUSG00000032373	carbonic anyhydrase 12 [Source:MGI Symbol;Acc:MGI:1923709]	1.9974507	2
Cbr2	11	120729489	120732114	-	0.0260286460	ENSMUSG00000025150	carbonyl reductase 2 [Source:MGI Symbol;Acc:MGI:107200]	-1.3971673	1
Ccdc135	8	95055103	95078141	+	0.0009762756	ENSMUSG00000031786	coiled-coil domain containing 135 [Source:MGI Symbol;Acc:MGI:2685616]	1.72536998	2
Cdhr4	9	107983287	107999684	+	0.0226657330	ENSMUSG00000032595	cadherin-related family member 4 [Source:MGI Symbol;Acc:MGI:1916648]	1.0202477	2
Cdkn1c	7	143458350	143461050	-	0.0427419770	ENSMUSG00000037664	cyclin-dependent kinase inhibitor 1C (P57) [Source:MGI Symbol;Acc:MGI:104564]	0.666692	2
Chrna2	14	66140960	66152948	+	0.0414713770	ENSMUSG00000022041	cholinergic receptor, nicotinic, alpha polypeptide 2 (neuronal) [Source:MGI Symbol;Acc:MGI:87886]	-2.21204015	1
Cldn2	X	139800828	139811386	+	0.0000312822	ENSMUSG00000047230	claudin 2 [Source:MGI Symbol;Acc:MGI:1276110]	3.3175542	2
Clic6	16	92485736	92541243	+	0.0000000050	ENSMUSG00000022949	chloride intracellular channel 6 [Source:MGI Symbol;Acc:MGI:2146607]	1.5429803	2
Col16a1	4	130047840	130099283	+	0.0014495755	ENSMUSG00000040690	collagen, type XVI, alpha 1 [Source:MGI Symbol;Acc:MGI:1095396]	-0.6551482	1
Col18a1	10	77052178	77166548	-	0.0007324343	ENSMUSG00000001435	collagen, type XVIII, alpha 1 [Source:MGI Symbol;Acc:MGI:88451]	-0.745678	1
Col27a1	4	63214004	63334991	+	0.0009762756	ENSMUSG00000045672	collagen, type XXVII, alpha 1 [Source:MGI Symbol;Acc:MGI:2672118]	-1.0699353	1
Col8a2	4	126286793	126314330	+	0.0302835440	ENSMUSG00000056174	collagen, type VIII, alpha 2 [Source:MGI Symbol;Acc:MGI:88464]	1.0708759	2
Col9a3	2	180597790	180622189	+	0.0287750600	ENSMUSG00000027570	collagen, type IX, alpha 3 [Source:MGI Symbol;Acc:MGI:894686]	0.5999494	2
CR536618.1	X	170009659	170019281	+	0.0031848343	ENSMUSG00000095562	No description	-0.5788993	1
Cyp26b1	6	84571414	84593908	-	0.0266405510	ENSMUSG00000063415	cytochrome P450, family 26, subfamily b, polypeptide 1 [Source:MGI Symbol;Acc:MGI:2176159]	-0.8726003	1
Cyp2a5	7	26835305	26843548	+	0.0000037273	ENSMUSG00000005547	cytochrome P450, family 2, subfamily a, polypeptide 5 [Source:MGI Symbol;Acc:MGI:88597]	-3.0246748	1
Ddc	11	11814101	11898144	-	0.0108018190	ENSMUSG00000020182	dopa decarboxylase [Source:MGI Symbol;Acc:MGI:94876]	-0.5403733	1
Dio2	12	90724554	90738438	-	0.0002221928	ENSMUSG00000007682	deiodinase, iodothyronine, type II [Source:MGI Symbol;Acc:MGI:1338833]	0.8895602	2
Dmrta2	4	109978053	109983687	+	0.0264056100	ENSMUSG00000047143	doublesex and mab-3 related transcription factor like family A2 [Source:MGI Symbol;Acc:MGI:2653629]	0.9501289	2
Ebf4	2	130295169	130370481	+	0.0473065400	ENSMUSG00000053552	early B cell factor 4 [Source:MGI Symbol;Acc:MGI:2385972]	-0.5685613	1
Eln	5	134703781	134747241	-	0.0378249320	ENSMUSG00000029675	elastin [Source:MGI Symbol;Acc:MGI:95317]	-1.0177414	1
Enpp2	15	54838898	54920146	-	0.0000000044	ENSMUSG00000022425	ectonucleotide pyrophosphatase/phosphodiesterase 2 [Source:MGI Symbol;Acc:MGI:1321390]	1.077356	2
Epha4	1	77367185	77515088	-	0.0045550280	ENSMUSG00000026235	Eph receptor A4 [Source:MGI Symbol;Acc:MGI:98277]	0.585893	2
Eps8l1	7	4460674	4479404	+	0.0009574503	ENSMUSG00000006154	EPS8-like 1 [Source:MGI Symbol;Acc:MGI:1914675]	-0.7698846	1
Eps8l2	7	141338880	141363020	+	0.0436774460	ENSMUSG00000025504	EPS8-like 2 [Source:MGI Symbol;Acc:MGI:2138828]	0.5323163	2
F5	1	164151838	164220277	+	0.0000000013	ENSMUSG00000026579	coagulation factor V [Source:MGI Symbol;Acc:MGI:88382]	3.1785275	2
Fat2	11	55250609	55336564	-	0.0000772055	ENSMUSG00000055333	FAT tumor suppressor homolog 2 (Drosophila) [Source:MGI Symbol;Acc:MGI:2685369]	-4.11404973	1
Fetub	16	22918382	22939766	+	0.0110166440	ENSMUSG00000022871	fetuin beta [Source:MGI Symbol;Acc:MGI:1890221]	-3.16892031	1
Folr1	7	101858331	101870788	-	0.0000000000	ENSMUSG00000001827	folate receptor 1 (adult) [Source:MGI Symbol;Acc:MGI:95568]	3.6595368	2
Gabra4	5	71569734	71658308	-	0.0000040955	ENSMUSG00000029211	gamma-aminobutyric acid (GABA) A receptor, subunit alpha 4 [Source:MGI Symbol;Acc:MGI:95616]	0.945456	2
Gm15564	16	35966752	35983230	-	0.0106343730	ENSMUSG00000086324	predicted gene 15564 [Source:MGI Symbol;Acc:MGI:3783013]	0.8060295	2
Gm15996	12	111730094	111731831	-	0.0138615780	ENSMUSG00000090026	predicted gene 15996 [Source:MGI Symbol;Acc:MGI:3802107]	-0.9949123	1
Gm17167	4	42206998	42235313	+	0.0281748880	ENSMUSG00000091542	predicted gene 17167 [Source:MGI Symbol;Acc:MGI:4937994]	-0.53065	1
Gm20481	17	34969912	34972136	+	0.0250108930	ENSMUSG00000092609	predicted gene 20481 [Source:MGI Symbol;Acc:MGI:5141946]	0.8456058	2
Gyltl1b	2	92365046	92374869	-	0.0000003522	ENSMUSG00000040434	glycosyltransferase-like 1B [Source:MGI Symbol;Acc:MGI:2443769]	2.62718983	2
Hcrt	11	100761069	100762931	-	0.0000000000	ENSMUSG00000045471	hypocretin [Source:MGI Symbol;Acc:MGI:1202306]	2.3169382	2
Hmgb2	8	57511843	57515999	+	0.0293398280	ENSMUSG00000054717	high mobility group box 2 [Source:MGI Symbol;Acc:MGI:96157]	-0.9220399	1
Hoxb3	11	96323326	96346685	+	0.0048173796	ENSMUSG00000048763	homeobox B3 [Source:MGI Symbol;Acc:MGI:96184]	-3.4597291	1
Hoxb3os	11	96345481	96354691	-	0.0305417520	ENSMUSG00000084844	homeobox B3, opposite strand transcript [Source:MGI Symbol;Acc:MGI:103211]	-3.4649205	1
Hoxb5	11	96302595	96306120	+	0.0401955020	ENSMUSG00000038700	homeobox B5 [Source:MGI Symbol;Acc:MGI:96186]	-3.4323529	1
Hoxc4	15	103018936	103036843	+	0.0266405510	ENSMUSG00000075394	homeobox C4 [Source:MGI Symbol;Acc:MGI:96195]	-3.51792595	1
Hps1	19	42755105	42779978	-	0.0008045358	ENSMUSG00000025188	Hermansky-Pudlak syndrome 1 homolog (human) [Source:MGI Symbol;Acc:MGI:2177763]	-0.6709624	1
Igfbp2	1	72824503	72852474	+	0.0001162401	ENSMUSG00000039323	insulin-like growth factor binding protein 2 [Source:MGI Symbol;Acc:MGI:96437]	0.8000598	2
Igsf5	16	96361668	96525580	+	0.0024257617	ENSMUSG00000000159	immunoglobulin superfamily, member 5 [Source:MGI Symbol;Acc:MGI:1919308]	0.5320684	2
Irx2	13	72628820	72634196	+	0.0001225725	ENSMUSG00000001504	Iroquois related homeobox 2 (Drosophila) [Source:MGI Symbol;Acc:MGI:1197526]	-1.1428525	1
Iyd	10	3540240	3554877	+	0.0107209790	ENSMUSG00000019762	iodotyrosine deiodinase [Source:MGI Symbol;Acc:MGI:1917587]	2.2578586	2
Kcne2	16	92292389	92298129	+	0.0000000081	ENSMUSG00000039672	potassium voltage-gated channel, Isk-related subfamily, gene 2 [Source:MGI Symbol;Acc:MGI:1891123]	5.3830339	2
Kcnj13	1	87386363	87394729	-	0.0000000183	ENSMUSG00000079436	potassium inwardly-rectifying channel, subfamily J, member 13 [Source:MGI Symbol;Acc:MGI:3781032]	3.8195206	2
Kitl	10	100015630	100100413	+	0.0002506630	ENSMUSG00000019966	kit ligand [Source:MGI Symbol;Acc:MGI:96974]	0.864494	2
Kl	5	150952607	150993809	+	0.0000000000	ENSMUSG00000058488	klotho [Source:MGI Symbol;Acc:MGI:1101771]	2.2199721	2
Lama1	17	67697265	67822645	+	0.0382044900	ENSMUSG00000032796	laminin, alpha 1 [Source:MGI Symbol;Acc:MGI:99892]	-0.9025492	1
Lars2	9	123366940	123462664	+	0.0044259857	ENSMUSG00000035202	leucyl-tRNA synthetase, mitochondrial [Source:MGI Symbol;Acc:MGI:2142973]	0.660776	2
Lbp	2	158306493	158332852	+	0.0130824870	ENSMUSG00000016024	lipopolysaccharide binding protein [Source:MGI Symbol;Acc:MGI:1098776]	0.8955857	2
Lef1	3	131110471	131224356	+	0.0204209180	ENSMUSG00000027985	lymphoid enhancer binding factor 1 [Source:MGI Symbol;Acc:MGI:96770]	0.5934993	2
Leng8	7	4137039	4148173	+	0.0001094430	ENSMUSG00000035545	leukocyte receptor cluster (LRC) member 8 [Source:MGI Symbol;Acc:MGI:2142195]	-0.643245	1
Lrrtm1	6	77242689	77257791	+	0.0078203090	ENSMUSG00000060780	leucine rich repeat transmembrane neuronal 1 [Source:MGI Symbol;Acc:MGI:2389173]	0.580738	2
Mab21l2	3	86545581	86548283	-	0.0012077800	ENSMUSG00000057777	mab-21-like 2 (C. elegans) [Source:MGI Symbol;Acc:MGI:1346022]	-1.81109085	1
Mapk13	17	28769307	28778698	+	0.0009762756	ENSMUSG00000004864	mitogen-activated protein kinase 13 [Source:MGI Symbol;Acc:MGI:1346864]	-2.8152694	1
Mas1	17	12841079	12868143	-	0.0001225725	ENSMUSG00000068037	MAS1 oncogene [Source:MGI Symbol;Acc:MGI:96918]	1.50402707	2
Mef2c	13	83504034	83667079	+	0.0287750600	ENSMUSG00000005583	myocyte enhancer factor 2C [Source:MGI Symbol;Acc:MGI:99458]	0.524341	2
Meg3	12	109541001	109571726	+	0.0482861300	ENSMUSG00000021268	maternally expressed 3 [Source:MGI Symbol;Acc:MGI:1202886]	-0.557163	1
Mid1	X	169685199	170005736	+	0.0391883500	ENSMUSG00000035299	midline 1 [Source:MGI Symbol;Acc:MGI:1100537]	-0.5090955	1
Mrap2	9	87144306	87184045	+	0.0004358072	ENSMUSG00000042761	melanocortin 2 receptor accessory protein 2 [Source:MGI Symbol;Acc:MGI:3609239]	-0.699694	1
Msx1	5	37820485	37824583	-	0.0134052650	ENSMUSG00000048450	homeobox, msh-like 1 [Source:MGI Symbol;Acc:MGI:97168]	1.723836335	2
Mt1	8	94179089	94180325	+	0.0415671130	ENSMUSG00000031765	metallothionein 1 [Source:MGI Symbol;Acc:MGI:97171]	-0.457632	1
Mt2	8	94172618	94173567	+	0.0158841310	ENSMUSG00000031762	metallothionein 2 [Source:MGI Symbol;Acc:MGI:97172]	-0.603719	1
Myo5b	18	74440936	74771493	+	0.0120894560	ENSMUSG00000025885	myosin VB [Source:MGI Symbol;Acc:MGI:106598]	0.618371	2
Neat1	19	5824708	5845478	-	0.0004140011	ENSMUSG00000092274	nuclear paraspeckle assembly transcript 1 (non-protein coding) [Source:MGI Symbol;Acc:MGI:1914211]	-0.577607	1
Neurod6	6	55677818	55681263	-	0.0101085810	ENSMUSG00000037984	neurogenic differentiation 6 [Source:MGI Symbol;Acc:MGI:106593]	1.50030642	2
Ntng1	3	109780040	110144011	-	0.0204209180	ENSMUSG00000059857	netrin G1 [Source:MGI Symbol;Acc:MGI:1934028]	0.4679957	2
Oca2	7	56239760	56536517	+	0.0031835462	ENSMUSG00000030450	oculocutaneous albinism II [Source:MGI Symbol;Acc:MGI:97454]	3.8778461	2
Omp	7	98143360	98145502	-	0.0228995420	ENSMUSG00000074006	olfactory marker protein [Source:MGI Symbol;Acc:MGI:97436]	-1.9453935	1
Otp	13	94875627	94883681	+	0.0190518600	ENSMUSG00000021685	orthopedia homolog (Drosophila) [Source:MGI Symbol;Acc:MGI:99835]	-1.1180214	1
Otx2	14	48657679	48667644	-	0.0056804237	ENSMUSG00000021848	orthodenticle homolog 2 (Drosophila) [Source:MGI Symbol;Acc:MGI:97451]	0.75791	2
Oxt	2	130576173	130577054	+	0.0378249320	ENSMUSG00000027301	oxytocin [Source:MGI Symbol;Acc:MGI:97453]	-1.2783847	1
Pcp4	16	96467606	96525793	+	0.0060232957	ENSMUSG00000090223	Purkinje cell protein 4 [Source:MGI Symbol;Acc:MGI:97509]	0.5262227	2
Pdlim2	14	70164218	70177681	-	0.0192252430	ENSMUSG00000022090	PDZ and LIM domain 2 [Source:MGI Symbol;Acc:MGI:2384850]	0.4758668	2
Pdp1	4	11958184	11966452	-	0.0000414545	ENSMUSG00000049225	pyruvate dehyrogenase phosphatase catalytic subunit 1 [Source:MGI Symbol;Acc:MGI:2685870]	0.637417	2
Pitx2	3	129199878	129219591	+	0.0052395530	ENSMUSG00000028023	paired-like homeodomain transcription factor 2 [Source:MGI Symbol;Acc:MGI:109340]	0.9198455	2
Pla2g5	4	138799244	138863482	-	0.0473065400	ENSMUSG00000041193	phospholipase A2, group V [Source:MGI Symbol;Acc:MGI:101899]	1.48649624	2
Plekhg1	10	3740364	3967303	+	0.0008668778	ENSMUSG00000040624	pleckstrin homology domain containing, family G (with RhoGef domain) member 1 [Source:MGI Symbol;Acc:MGI:2676551]	0.5971285	2
Pmch	10	88091072	88092375	+	0.0000000115	ENSMUSG00000035383	pro-melanin-concentrating hormone [Source:MGI Symbol;Acc:MGI:97629]	0.8917327	2
Prg4	1	150449412	150466165	-	0.0070437337	ENSMUSG00000006014	proteoglycan 4 (megakaryocyte stimulating factor, articular superficial zone protein) [Source:MGI Symbol;Acc:MGI:1891344]	-0.7584136	1
Prkcd	14	30595356	30626210	-	0.0000000026	ENSMUSG00000021948	protein kinase C, delta [Source:MGI Symbol;Acc:MGI:97598]	1.149431	2
Prlr	15	10177238	10349180	+	0.0000597152	ENSMUSG00000005268	prolactin receptor [Source:MGI Symbol;Acc:MGI:97763]	0.971011	2
Ptk2b	14	66153257	66281052	-	0.0293398280	ENSMUSG00000059456	PTK2 protein tyrosine kinase 2 beta [Source:MGI Symbol;Acc:MGI:104908]	0.5047179	2
Ptpn3	4	57190841	57301837	-	0.0002754160	ENSMUSG00000038764	protein tyrosine phosphatase, non-receptor type 3 [Source:MGI Symbol;Acc:MGI:105307]	0.6677947	2
Ptpn4	1	119658044	119837613	-	0.0048173796	ENSMUSG00000026384	protein tyrosine phosphatase, non-receptor type 4 [Source:MGI Symbol;Acc:MGI:1099792]	0.6885826	2
Ramp3	11	6658521	6677475	+	0.0045157840	ENSMUSG00000041046	receptor (calcitonin) activity modifying protein 3 [Source:MGI Symbol;Acc:MGI:1860292]	0.740486	2
Rasgrp1	2	117279993	117343001	-	0.0000283933	ENSMUSG00000027347	RAS guanyl releasing protein 1 [Source:MGI Symbol;Acc:MGI:1314635]	0.725169	2
Rbm47	5	66016549	66173117	-	0.0033887096	ENSMUSG00000070780	RNA binding motif protein 47 [Source:MGI Symbol;Acc:MGI:2384294]	1.2260877	2
Rgs11	17	26202951	26211324	+	0.0101085810	ENSMUSG00000024186	regulator of G-protein signaling 11 [Source:MGI Symbol;Acc:MGI:1354739]	-0.6654016	1
Rgs16	1	153740349	153745468	+	0.0491564700	ENSMUSG00000026475	regulator of G-protein signaling 16 [Source:MGI Symbol;Acc:MGI:108407]	0.5621586	2
Rgs4	1	169741477	169747642	-	0.0001225725	ENSMUSG00000038530	regulator of G-protein signaling 4 [Source:MGI Symbol;Acc:MGI:108409]	0.683003	2
Rora	9	68653786	69388246	+	0.0024390756	ENSMUSG00000032238	RAR-related orphan receptor alpha [Source:MGI Symbol;Acc:MGI:104661]	0.6315403	2
Rtel1	2	181319739	181356616	+	0.0336381980	ENSMUSG00000038685	regulator of telomere elongation helicase 1 [Source:MGI Symbol;Acc:MGI:2139369]	-0.5721997	1
Sh3d19	3	86084434	86130522	+	0.0120894560	ENSMUSG00000028082	SH3 domain protein D19 [Source:MGI Symbol;Acc:MGI:1350923]	0.6260491	2
Shank3	15	89499623	89560261	+	0.0007280641	ENSMUSG00000022623	SH3/ankyrin domain gene 3 [Source:MGI Symbol;Acc:MGI:1930016]	0.531843	2
Slc10a4	5	73006904	73014683	+	0.0052322075	ENSMUSG00000029219	solute carrier family 10 (sodium/bile acid cotransporter family), member 4 [Source:MGI Symbol;Acc:MGI:3606480]	-0.9287448	1
Slc16a11	11	70212752	70216417	+	0.0019264042	ENSMUSG00000040938	solute carrier family 16 (monocarboxylic acid transporters), member 11 [Source:MGI Symbol;Acc:MGI:2663709]	-0.6031827	1
Slc16a6	11	109450855	109473598	-	0.0056804237	ENSMUSG00000041920	solute carrier family 16 (monocarboxylic acid transporters), member 6 [Source:MGI Symbol;Acc:MGI:2144585]	-0.645428	1
Slc4a5	6	83219828	83304945	+	0.0000000000	ENSMUSG00000068323	solute carrier family 4, sodium bicarbonate cotransporter, member 5 [Source:MGI Symbol;Acc:MGI:2443220]	2.90482387	2
Slc6a20a	9	123636122	123678885	-	0.0301960220	ENSMUSG00000036814	solute carrier family 6 (neurotransmitter transporter), member 20A [Source:MGI Symbol;Acc:MGI:2143217]	-0.6142177	1
Slc6a4	11	76998603	77032340	+	0.0000040955	ENSMUSG00000020838	solute carrier family 6 (neurotransmitter transporter, serotonin), member 4 [Source:MGI Symbol;Acc:MGI:96285]	-3.7131751	1
Slco1a5	6	142234227	142322981	-	0.0002754160	ENSMUSG00000063975	solute carrier organic anion transporter family, member 1a5 [Source:MGI Symbol;Acc:MGI:1351865]	2.00359977	2
Sostdc1	12	36314169	36318452	+	0.0078058740	ENSMUSG00000036169	sclerostin domain containing 1 [Source:MGI Symbol;Acc:MGI:1913292]	1.7221886	2
Sphk1	11	116530925	116536674	+	0.0001530622	ENSMUSG00000061878	sphingosine kinase 1 [Source:MGI Symbol;Acc:MGI:1316649]	-0.840375	1
Spp1	5	104435118	104441050	+	0.0001088649	ENSMUSG00000029304	secreted phosphoprotein 1 [Source:MGI Symbol;Acc:MGI:98389]	-0.8126124	1
Stil	4	115000159	115043196	+	0.0365549850	ENSMUSG00000028718	Scl/Tal1 interrupting locus [Source:MGI Symbol;Acc:MGI:107477]	1.8629674	2
Sulf1	1	12692430	12860371	+	0.0001881928	ENSMUSG00000016918	sulfatase 1 [Source:MGI Symbol;Acc:MGI:2138563]	0.7501998	2
Synpo2	3	123076519	123236149	-	0.0000023273	ENSMUSG00000050315	synaptopodin 2 [Source:MGI Symbol;Acc:MGI:2153070]	0.905304	2
Sytl4	X	133936385	133981812	-	0.0427419770	ENSMUSG00000031255	synaptotagmin-like 4 [Source:MGI Symbol;Acc:MGI:1351606]	-0.5838936	1
Th	7	142892752	142931128	-	0.0006316462	ENSMUSG00000000214	tyrosine hydroxylase [Source:MGI Symbol;Acc:MGI:98735]	-0.673157	1
Thbs3	3	89215180	89226837	+	0.0059073633	ENSMUSG00000028047	thrombospondin 3 [Source:MGI Symbol;Acc:MGI:98739]	-0.6634765	1
Tle2	10	81574561	81590845	+	0.0293398280	ENSMUSG00000034771	transducin-like enhancer of split 2, homolog of Drosophila E(spl) [Source:MGI Symbol;Acc:MGI:104635]	-0.564693	1
Tmem38b	4	53826045	53862019	+	0.0005952520	ENSMUSG00000028420	transmembrane protein 38B [Source:MGI Symbol;Acc:MGI:1098718]	0.9230905	2
Tmem72	6	116692630	116716913	-	0.0036505444	ENSMUSG00000048108	transmembrane protein 72 [Source:MGI Symbol;Acc:MGI:2442707]	2.8345226	2
Tmprss7	16	45656315	45693658	-	0.0101679190	ENSMUSG00000033177	transmembrane serine protease 7 [Source:MGI Symbol;Acc:MGI:2686594]	-1.60791027	1
Tnnt1	7	4504570	4516382	-	0.0036463880	ENSMUSG00000064179	troponin T1, skeletal, slow [Source:MGI Symbol;Acc:MGI:1333868]	0.589565	2
Tnnt3	7	142498836	142516009	+	0.0134234310	ENSMUSG00000061723	troponin T3, skeletal, fast [Source:MGI Symbol;Acc:MGI:109550]	-1.1428694	1
Trpv4	5	114622152	114658421	-	0.0000000174	ENSMUSG00000014158	transient receptor potential cation channel, subfamily V, member 4 [Source:MGI Symbol;Acc:MGI:1926945]	1.4654407	2
Ttll6	11	96133786	96165451	+	0.0065005305	ENSMUSG00000038756	tubulin tyrosine ligase-like family, member 6 [Source:MGI Symbol;Acc:MGI:2683461]	-1.7401999	1
Ttr	18	20665250	20674324	+	0.0000000000	ENSMUSG00000061808	transthyretin [Source:MGI Symbol;Acc:MGI:98865]	4.9881163	2
Vav3	3	109340653	109685698	+	0.0005332028	ENSMUSG00000033721	vav 3 oncogene [Source:MGI Symbol;Acc:MGI:1888518]	0.6819978	2
Wdr86	5	24711738	24730727	-	0.0048173796	ENSMUSG00000055235	WD repeat domain 86 [Source:MGI Symbol;Acc:MGI:1915466]	1.7264456	2
Wfdc2	2	164562413	164568510	+	0.0008257816	ENSMUSG00000017723	WAP four-disulfide core domain 2 [Source:MGI Symbol;Acc:MGI:1914951]	3.34117612	2

Supplementary Table 2

37 genes DE in MPTP lesioned mice which showed a degree of recovery upon cu(atsm) treatment

	Fold change normalised to MPTP-SSV				
**Probe**	**WT-SSV**	**WT-Cu(atsm)**	**MPTP-Cu(atsm)**	**MPTP-SSV**	**Description**
Abca4	0.36627	0.409101	0.562904	1	ATP-binding cassette, sub-family A (ABC1), member 4
Ace	0.662706	0.676007	0.758681	1	angiotensin I converting enzyme (peptidyl-dipeptidase A)
Adamts10	1.68779	1.51809	1.25604	1	a disintegrin-like and metallopeptidase (reprolysin type) with thrombospondin type 1 motif, 10
Calml4	0.305621	0.260284	0.441061	1	calmodulin-like 4
Car12	0.250442	0.250658	0.449658	1	carbonic anyhydrase 12
Ccdc135	0.302421	0.285935	0.53137	1	coiled-coil domain containing 135
Clic6	0.343176	0.352853	0.601273	1	chloride intracellular channel 6
Col16a1	1.57478	1.32256	1.21661	1	collagen, type XVI, alpha 1
Ddc	1.45435	2.07567	1.36992	1	dopa decarboxylase
Enpp2	0.473897	0.498761	0.746366	1	ectonucleotide pyrophosphatase/phosphodiesterase 2
F5	0.110451	0.091492	0.436015	1	coagulation factor V
Folr1	0.0791352	0.1322	0.375228	1	folate receptor 1 (adult)
Hcrt	0.200693	0.46763	0.560013	1	hypocretin [
Kcne2	0.0239632	0.0746921	0.513604	1	potassium voltage-gated channel, Isk-related subfamily, gene 2
Kl	0.214646	0.219168	0.478185	1	klotho
Leng8	1.56184	1.48208	1.20297	1	leukocyte receptor cluster (LRC) member 8
Lrrtm1	0.668622	0.638938	0.827055	1	leucine rich repeat transmembrane neuronal 1
Mt1	1.37329	1.4577	1.24974	1	metallothionein 1
Mt2	1.51963	1.4747	1.23985	1	metallothionein 2
Neat1	1.49237	1.60687	1.28945	1	nuclear paraspeckle assembly transcript 1 (non-protein coding)
Prg4	1.69163	3.79471	1.42879	1	proteoglycan 4 (megakaryocyte stimulating factor, articular superficial zone protein)
Prkcd	0.450803	0.500607	0.780134	1	protein kinase C, delta
Ptk2b	0.704798	0.729535	0.668057	1	PTK2 protein tyrosine kinase 2 beta
Ptpn3	0.629468	0.595304	0.835626	1	protein tyrosine phosphatase, non-receptor type 3
Ramp3	0.598538	0.541381	0.789953	1	receptor (calcitonin) activity modifying protein 3
Rasgrp1	0.604927	0.633724	0.825488	1	RAS guanyl releasing protein 1
Rgs11	1.58601	1.46885	1.22889	1	regulator of G-protein signaling 11
Slc10a4	1.90362	2.37891	1.63281	1	solute carrier family 10 (sodium/bile acid cotransporter family), member 4
Slc4a5	0.133524	0.132854	0.537155	1	solute carrier family 4, sodium bicarbonate cotransporter, member 5
Sostdc1	0.303089	0.212827	0.505771	1	sclerostin domain containing 1
Sphk1	1.79051	2.04276	1.49529	1	sphingosine kinase 1
Spp1	1.75639	1.58298	1.24629	1	secreted phosphoprotein 1 [Source:MGI Symbol;Acc:MGI:98389]
Synpo2	0.53392	0.575823	0.847184	1	synaptopodin 2
Th	1.59456	2.45607	1.70024	1	tyrosine hydroxylase
Tmem38b	0.527378	0.52025	0.559967	1	transmembrane protein 38B .
Trpv4	0.362125	0.386409	0.643145	1	transient receptor potential cation channel, subfamily V, member 4
Ttr	0.0315085	0.034921	0.343926	1	transthyretin

Supplementary Table 3

DE genes in unlesioned mice treated with Cu(atsm) compared to unleisoned sham controls

Probe	Chromosome	Start	End	Strand	Diff p-value	ID	Description
Fn1	1	71585523	71653171	-	0.044272628	ENSMUSG00000026193	fibronectin 1 [Source:MGI Symbol;Acc:MGI:95566]
Igfbp2	1	72824503	72852474	+	0.001431661	ENSMUSG00000039323	insulin-like growth factor binding protein 2 [Source:MGI Symbol;Acc:MGI:96437]
Ecel1	1	87147655	87156521	-	0.001220296	ENSMUSG00000026247	endothelin converting enzyme-like 1 [Source:MGI Symbol;Acc:MGI:1343461]
En1	1	120602418	120609296	+	2.28E-07	ENSMUSG00000058665	engrailed 1 [Source:MGI Symbol;Acc:MGI:95389]
Fmod	1	134037254	134048277	+	1.24E-05	ENSMUSG00000041559	fibromodulin [Source:MGI Symbol;Acc:MGI:1328364]
Cfh	1	140085855	140183411	-	0.02271886	ENSMUSG00000026365	complement component factor h [Source:MGI Symbol;Acc:MGI:88385]
Prg4	1	150449412	150466165	-	0	ENSMUSG00000006014	proteoglycan 4 (megakaryocyte stimulating factor, articular superficial zone protein) [Source:MGI Symbol;Acc:MGI:1891344]
Myoc	1	162639150	162649693	+	5.75E-05	ENSMUSG00000026697	myocilin [Source:MGI Symbol;Acc:MGI:1202864]
Ptgds	2	25466709	25470046	-	2.12E-06	ENSMUSG00000015090	prostaglandin D2 synthase (brain) [Source:MGI Symbol;Acc:MGI:99261]
C130021I20Rik	2	33641193	33646349	+	0.006471183	ENSMUSG00000052951	Riken cDNA C130021I20 gene [Source:MGI Symbol;Acc:MGI:3639863]
Nr4a2	2	57106830	57124003	-	1.21E-04	ENSMUSG00000026826	nuclear receptor subfamily 4, group A, member 2 [Source:MGI Symbol;Acc:MGI:1352456]
Oxt	2	130576173	130577054	+	3.25E-05	ENSMUSG00000027301	oxytocin [Source:MGI Symbol;Acc:MGI:97453]
Avp	2	130580620	130582554	-	1.74E-04	ENSMUSG00000037727	arginine vasopressin [Source:MGI Symbol;Acc:MGI:88121]
6330527O06Rik	2	136052239	136069917	+	2.65E-04	ENSMUSG00000027270	RIKEN cDNA 6330527O06 gene [Source:MGI Symbol;Acc:MGI:1923411]
Gm14295	2	176798612	176811223	+	0.044272628	ENSMUSG00000078877	predicted gene 14295 [Source:MGI Symbol;Acc:MGI:3709624]
Ntrk1	3	87778244	87795162	-	0.012073561	ENSMUSG00000028072	neurotrophic tyrosine kinase, receptor, type 1 [Source:MGI Symbol;Acc:MGI:97383]
Pitx2	3	129199878	129219591	+	1.23E-11	ENSMUSG00000028023	paired-like homeodomain transcription factor 2 [Source:MGI Symbol;Acc:MGI:109340]
Slc26a7	4	14502430	14621805	-	0.006008672	ENSMUSG00000040569	solute carrier family 26, member 7 [Source:MGI Symbol;Acc:MGI:2384791]
1700045I11Rik	4	42170845	42171335	+	0.001010485	ENSMUSG00000096609	RIKEN cDNA 1700045I11 gene [Source:MGI Symbol;Acc:MGI:1920600]
Gm2500	4	42668043	42668438	+	0.001566385	ENSMUSG00000094731	predicted gene 2500 [Source:MGI Symbol;Acc:MGI:3780667]
Col27a1	4	63214004	63334991	+	4.12E-04	ENSMUSG00000045672	collagen, type XXVII, alpha 1 [Source:MGI Symbol;Acc:MGI:2672118]
Bnc2	4	84275095	84675275	-	0.002523582	ENSMUSG00000028487	basonuclin 2 [Source:MGI Symbol;Acc:MGI:2443805]
Lrrc17	5	21543527	21575900	+	0.04815859	ENSMUSG00000039883	leucine rich repeat containing 17 [Source:MGI Symbol;Acc:MGI:1921761]
Pcolce	5	137605103	137613784	-	0.025553491	ENSMUSG00000029718	procollagen C-endopeptidase enhancer protein [Source:MGI Symbol;Acc:MGI:105099]
Calcr	6	3685677	3764713	-	3.48E-05	ENSMUSG00000023964	calcitonin receptor [Source:MGI Symbol;Acc:MGI:101950]
Slc13a4	6	35267957	35308131	-	4.22E-07	ENSMUSG00000029843	solute carrier family 13 (sodium/sulfate symporters), member 4 [Source:MGI Symbol;Acc:MGI:2442367]
Dgki	6	36846022	37300184	-	0.038702004	ENSMUSG00000038665	diacylglycerol kinase, iota [Source:MGI Symbol;Acc:MGI:2443430]
Trh	6	92242061	92244650	-	0.002728222	ENSMUSG00000005892	thyrotropin releasing hormone [Source:MGI Symbol;Acc:MGI:98823]
Ret	6	118151748	118197744	-	0.003023582	ENSMUSG00000030110	ret proto-oncogene [Source:MGI Symbol;Acc:MGI:97902]
Slc6a13	6	121300227	121337733	+	0.007026591	ENSMUSG00000030108	solute carrier family 6 (neurotransmitter transporter, GABA), member 13 [Source:MGI Symbol;Acc:MGI:95629]
Slc6a12	6	121343076	121365775	+	8.63E-12	ENSMUSG00000030109	solute carrier family 6 (neurotransmitter transporter, betaine/GABA), member 12 [Source:MGI Symbol;Acc:MGI:95628]
Gucy2c	6	136697285	136781765	-	0.021095537	ENSMUSG00000042638	guanylate cyclase 2c [Source:MGI Symbol;Acc:MGI:106903]
Mgp	6	136872436	136875805	-	0.014821769	ENSMUSG00000030218	matrix Gla protein [Source:MGI Symbol;Acc:MGI:96976]
Pou2f2	7	25091909	25159922	-	0.03626019	ENSMUSG00000008496	POU domain, class 2, transcription factor 2 [Source:MGI Symbol;Acc:MGI:101897]
Cyp2g1	7	26808927	26821197	+	0.013326621	ENSMUSG00000049685	cytochrome P450, family 2, subfamily g, polypeptide 1 [Source:MGI Symbol;Acc:MGI:109612]
Cyp2a5	7	26835305	26843548	+	4.76E-08	ENSMUSG00000005547	cytochrome P450, family 2, subfamily a, polypeptide 5 [Source:MGI Symbol;Acc:MGI:88597]
Dmkn	7	30763756	30781066	+	0.021095537	ENSMUSG00000060962	dermokine [Source:MGI Symbol;Acc:MGI:1920962]
Fxyd5	7	31032722	31042481	-	0.023287956	ENSMUSG00000009687	FXYD domain-containing ion transport regulator 5 [Source:MGI Symbol;Acc:MGI:1201785]
AC149868.1	7	45176349	45179597	-	0.004332969	ENSMUSG00000095276	uncharacterized protein LOC210541 precursor [Source:RefSeq peptide;Acc:NP_001182184]
Adamtsl3	7	82335694	82614450	+	0.004587912	ENSMUSG00000070469	ADAMTS-like 3 [Source:MGI Symbol;Acc:MGI:3028499]
Tnnt3	7	142498836	142516009	+	3.73E-06	ENSMUSG00000061723	troponin T3, skeletal, fast [Source:MGI Symbol;Acc:MGI:109550]
Igf2	7	142650766	142666816	-	8.29E-06	ENSMUSG00000048583	insulin-like growth factor 2 [Source:MGI Symbol;Acc:MGI:96434]
Th	7	142892752	142931128	-	8.27E-07	ENSMUSG00000000214	tyrosine hydroxylase [Source:MGI Symbol;Acc:MGI:98735]
Chrnb3	8	27368711	27399729	+	1.15E-12	ENSMUSG00000031492	cholinergic receptor, nicotinic, beta polypeptide 3 [Source:MGI Symbol;Acc:MGI:106212]
Chrna6	8	27403214	27413944	-	0	ENSMUSG00000031491	cholinergic receptor, nicotinic, alpha polypeptide 6 [Source:MGI Symbol;Acc:MGI:106213]
Cdh1	8	106603351	106670246	+	2.03E-04	ENSMUSG00000000303	cadherin 1 [Source:MGI Symbol;Acc:MGI:88354]
Aldh1a2	9	71215789	71296243	+	0.003023582	ENSMUSG00000013584	aldehyde dehydrogenase family 1, subfamily A2 [Source:MGI Symbol;Acc:MGI:107928]
Unc13c	9	73480870	73933567	-	0.047703236	ENSMUSG00000062151	unc-13 homolog C (C. elegans) [Source:MGI Symbol;Acc:MGI:2149021]
Mrap2	9	87144306	87184045	+	0.0225864	ENSMUSG00000042761	melanocortin 2 receptor accessory protein 2 [Source:MGI Symbol;Acc:MGI:3609239]
Zic1	9	91358058	91365810	-	5.23E-04	ENSMUSG00000032368	zinc finger protein of the cerebellum 1 [Source:MGI Symbol;Acc:MGI:106683]
Zic4	9	91362413	91389348	+	0.036677167	ENSMUSG00000036972	zinc finger protein of the cerebellum 4 [Source:MGI Symbol;Acc:MGI:107201]
Lars2	9	123366940	123462664	+	1.53E-07	ENSMUSG00000035202	leucyl-tRNA synthetase, mitochondrial [Source:MGI Symbol;Acc:MGI:2142973]
Sgk1	10	21882184	21999903	+	3.16E-07	ENSMUSG00000019970	serum/glucocorticoid regulated kinase 1 [Source:MGI Symbol;Acc:MGI:1340062]
Tbata	10	61171954	61188841	+	9.04E-04	ENSMUSG00000020096	thymus, brain and testes associated [Source:MGI Symbol;Acc:MGI:1923820]
Col6a2	10	76595762	76623630	-	0.044272628	ENSMUSG00000020241	collagen, type VI, alpha 2 [Source:MGI Symbol;Acc:MGI:88460]
Col18a1	10	77052178	77166548	-	8.19E-04	ENSMUSG00000001435	collagen, type XVIII, alpha 1 [Source:MGI Symbol;Acc:MGI:88451]
Aire	10	78030022	78043610	-	0.023260545	ENSMUSG00000000731	autoimmune regulator (autoimmune polyendocrinopathy candidiasis ectodermal dystrophy) [Source:MGI Symbol;Acc:MGI:1338803]
Pmch	10	88091072	88092375	+	3.34E-06	ENSMUSG00000035383	pro-melanin-concentrating hormone [Source:MGI Symbol;Acc:MGI:97629]
4930547N16Rik	10	88091432	88146941	-	2.07E-06	ENSMUSG00000035365	RIKEN cDNA 4930547N16 gene [Source:MGI Symbol;Acc:MGI:1922567]
Ddc	11	11814101	11898144	-	4.02E-05	ENSMUSG00000020182	dopa decarboxylase [Source:MGI Symbol;Acc:MGI:94876]
Slc47a1	11	61343401	61378345	-	0.009349126	ENSMUSG00000010122	solute carrier family 47, member 1 [Source:MGI Symbol;Acc:MGI:1914723]
Slc6a4	11	76998603	77032340	+	0.016081538	ENSMUSG00000020838	solute carrier family 6 (neurotransmitter transporter, serotonin), member 4 [Source:MGI Symbol;Acc:MGI:96285]
Hcrt	11	100761069	100762931	-	7.19E-13	ENSMUSG00000045471	hypocretin [Source:MGI Symbol;Acc:MGI:1202306]
Mrc2	11	105292643	105351139	+	0.022493869	ENSMUSG00000020695	mannose receptor, C type 2 [Source:MGI Symbol;Acc:MGI:107818]
Cbr2	11	120729489	120732114	-	0.04711677	ENSMUSG00000025150	carbonyl reductase 2 [Source:MGI Symbol;Acc:MGI:107200]
4921506M07Rik	12	57564113	57737928	+	5.20E-04	ENSMUSG00000046782	RIKEN cDNA 4921506M07 gene [Source:MGI Symbol;Acc:MGI:1918096]
Slc6a3	13	73536747	73578672	+	0	ENSMUSG00000021609	solute carrier family 6 (neurotransmitter transporter, dopamine), member 3 [Source:MGI Symbol;Acc:MGI:94862]
Cartpt	13	99898484	99900683	-	1.50E-08	ENSMUSG00000021647	CART prepropeptide [Source:MGI Symbol;Acc:MGI:1351330]
Lgals3	14	47367751	47386160	+	0.010593314	ENSMUSG00000050335	lectin, galactose binding, soluble 3 [Source:MGI Symbol;Acc:MGI:96778]
Nov	15	54745702	54754039	+	4.51E-05	ENSMUSG00000037362	nephroblastoma overexpressed gene [Source:MGI Symbol;Acc:MGI:109185]
Gm15564	16	35966752	35983230	-	0.007257836	ENSMUSG00000086324	predicted gene 15564 [Source:MGI Symbol;Acc:MGI:3783013]
Ncam2	16	81200697	81624285	+	0.023499245	ENSMUSG00000022762	neural cell adhesion molecule 2 [Source:MGI Symbol;Acc:MGI:97282]
Mas1	17	12841079	12868143	-	6.58E-06	ENSMUSG00000068037	MAS1 oncogene [Source:MGI Symbol;Acc:MGI:96918]
Mapk13	17	28769307	28778698	+	0.003579086	ENSMUSG00000004864	mitogen-activated protein kinase 13 [Source:MGI Symbol;Acc:MGI:1346864]
Six3	17	85613608	85631813	+	0.04961885	ENSMUSG00000038805	sine oculis-related homeobox 3 homolog (Drosophila) [Source:MGI Symbol;Acc:MGI:102764]
Prdm6	18	53464546	53575907	+	0.04711677	ENSMUSG00000069378	PR domain containing 6 [Source:MGI Symbol;Acc:MGI:2684938]
Myo5b	18	74440936	74771493	+	0.04711677	ENSMUSG00000025885	myosin VB [Source:MGI Symbol;Acc:MGI:106598]
Slc22a6	19	8617996	8628299	+	3.54E-04	ENSMUSG00000024650	solute carrier family 22 (organic anion transporter), member 6 [Source:MGI Symbol;Acc:MGI:892001]
Aldh1a1	19	20601961	20643462	+	4.38E-04	ENSMUSG00000053279	aldehyde dehydrogenase family 1, subfamily A1 [Source:MGI Symbol;Acc:MGI:1353450]
Slc18a2	19	59260878	59296012	+	7.67E-08	ENSMUSG00000025094	solute carrier family 18 (vesicular monoamine), member 2 [Source:MGI Symbol;Acc:MGI:106677]
4932411N23Rik	X	126812462	126834004	-	4.53E-04	ENSMUSG00000058670	RIKEN cDNA 4932411N23 gene [Source:MGI Symbol;Acc:MGI:3045322]
Sytl4	X	133936385	133981812	-	0.013229314	ENSMUSG00000031255	synaptotagmin-like 4 [Source:MGI Symbol;Acc:MGI:1351606]
Irs4	X	141710998	141725263	-	0.044485927	ENSMUSG00000054667	insulin receptor substrate 4 [Source:MGI Symbol;Acc:MGI:1338009]
CR536618.1	X	170009659	170019281	+	3.60E-06	ENSMUSG00000095562	No description
AC151712.4	Y	90784738	90816464	+	5.32E-07	ENSMUSG00000096768	erythroid differentiation regulator 1 [Source:RefSeq peptide;Acc:NP_579940]

Supplementary 4

Validation of fold expression changes between MPTP lesioned mice and upon treatment with cu(atsm) by qRT-PCR

Probe	WT-SSV	WT-Cu(atsm)	MPTP-Cu(atsm)
Abca4	0.36627	0.409101	0.562904
ABCA4	0.4252	0.2873	0.4685
Ace	0.662706	0.676007	0.758681
ACE	0.641	0.63	0.747
Car12	0.250442	0.250658	0.449658
CAR12	0.3433	0.2922	0.5268
Ddc	1.45435	2.07567	1.36992
DDC	1.591	2.0196	1.2732
Kl	0.214646	0.219168	0.478185
KL	0.3198	0.1358	0.4779
Lrrtm1	0.668622	0.638938	0.827055
LRRTM1	0.907	0.7647	0.9809
Mt1	1.37329	1.4577	1.24974
MT1	1.3276	1.6518	1.3825
Mt2	1.51963	1.4747	1.23985
MT2	1.1818	1.3456	1.0911
Prkcd	0.450803	0.500607	0.780134
PRKCD	0.7918	0.6119	0.9552
Ptk2b	0.704798	0.729535	0.668057
PTK2B	0.8616	1.5546	0.8492
Ptpn3	0.629468	0.595304	0.835626
PTPN3	0.8657	0.7651	0.9361
Ramp3	0.598538	0.541381	0.789953
RAMP3	0.8438	0.5525	0.5525
Synpo2	0.53392	0.575823	0.847184
SYNPO2	0.8605	0.6886	1.0003
Th	1.59456	2.45607	1.70024
TH	2.67	1.82	2.33
Ttr	0.0315085	0.034921	0.343926
TTR	0.2915	0.0206	0.5141

Gene names coloured in small capitals are fold changes obtained from deep sequencing study
Gene names coloured in all capitals are fold changes obtained from qRT-PCR study
All data are fold changes (linear) normalised to MPTP lesioned

Supplementary Table 5: Functional categories

Gene	Ensembl ID	Description	Gene Summary (provided by RefSeq/AceView/GeneCards)	Biological Functional Class	Gene Type	Log2 Fold-c	Gene cluster
1500015O10Rik	ENSMUSG00000026051	RIKEN cDNA 1500015O10 gene [Source:MGI Symbol;Acc:MGI:1926146]	Function is yet unknown.	Unknown	Unknown	3.10248	2
4930547N16Rik	ENSMUSG00000035365	RIKEN cDNA 4930547N16 gene [Source:MGI Symbol;Acc:MGI:1922567]	Official name: PARP1 binding protein (Parpbp). Parpbp required to suppress inappropriate homologous recombination, thereby playing a central role DNA repair and in the maintenance of genomic stability.	Transcription/transcription factor activity	Binding protein	0.896957	2
6330403A02Rik	ENSMUSG00000053963	RIKEN cDNA 6330403A02 gene [Source:MGI Symbol;Acc:MGI:2138735]	Function is yet unknown.	Unknown	Unknown	0.511204	2
6330527O06Rik	ENSMUSG00000027270	RIKEN cDNA 6330527O06 gene [Source:MGI Symbol;Acc:MGI:1923411]	Function is yet unknown. Other names: Protein C20orf103brain and dendritic cell-associated LAMP, or brain-associated LAMP-like protein.	Unknown	Unknown	-0.690673	1
Abca4	ENSMUSG00000028125	ATP-binding cassette, sub-family A (ABC1), member 4 [Source:MGI Symbol;Acc:MGI:109424]	This gene is a member of the superfamily of ATP-binding cassette (ABC) transporters. ABC proteins transport various molecules across extra- and intracellular membranes.	Transporter	Transport	1.449019	2
Abhd12b	ENSMUSG00000090121	abhydrolase domain containing 12B [Source:MGI Symbol;Acc:MGI:2685650]	Function not quite known yet. May involve in acylglycerol lipase activity.	Others	Unknown	0.807348	2
AC149868.1	ENSMUSG00000095276	uncharacterized protein LOC210541 precursor [Source:RefSeq peptide;Acc:NP_001182184]	Function is yet unknown.	Unknown	Unknown	-2.749345	1
AC151712.4	ENSMUSG00000096768	erythroid differentiation regulator 1 [Source:RefSeq peptide;Acc:NP_579940]	Function is yet unknown.	Unknown	Unknown	-0.588162	1
Ace	ENSMUSG00000020681	angiotensin I converting enzyme (peptidyl-dipeptidase A) 1 [Source:MGI Symbol;Acc:MGI:87874]	This gene encodes an enzyme involved in catalyzing the conversion of angiotensin I into a physiologically active peptide angiotensin II, this results in an increase of the vasoconstrictor activity of angiotensin.	Others	Enzyme	0.593558	2
Acrbp	ENSMUSG00000072770	proacrosin binding protein [Source:MGI Symbol;Acc:MGI:1859515]	The protein encoded by this gene may be involved in packaging and condensation of the acrosin zymogen in the acrosomal matrix via its association with proacrosin.	Others	Packaging	-0.928197	1
Adamts10	ENSMUSG00000024299	a disintegrin-like and metallopeptidase (reprolysin type) with thrombospondin type 1 motif, 10 [Source:MGI Symbol;Acc:MGI:2449112]	This gene belongs to the ADAMTS (a disintegrin and metalloproteinase domain with thrombospondin type-1 motifs) family of zinc-dependent proteases and participate in microfibrils assembly.	Structural constituent	Enzyme	-0.755133	1
Adcy1	ENSMUSG00000020431	adenylate cyclase 1 [Source:MGI Symbol;Acc:MGI:99677]	A member of the of adenylate cyclase gene family that is primarily expressed in the brain. This protein is regulated by calcium/calmodulin concentration and may be involved in brain development.	Enzyme regulator activity	Enzyme	0.673891	2
AI480526	ENSMUSG00000090086	expressed sequence AI480526 [Source:MGI Symbol;Acc:MGI:2140910]	Function is yet unknown.	Unknown	Unknown	-0.619136	1
Aqp1	ENSMUSG00000004655	aquaporin 1 [Source:MGI Symbol;Acc:MGI:103201]	Aquaporins are a family of small integral membrane proteins related to the major intrinsic protein. This gene encodes an aquaporin which functions as a molecular water channel protein.	Transporter	Channel	3.203067	2
Arrdc1	ENSMUSG00000026972	arrestin domain containing 1 [Source:MGI Symbol;Acc:MGI:2446136]	Function is yet unknown.	Others	Unknown	-0.56512	1
Atp2b1	ENSMUSG00000019943	ATPase, Ca++ transporting, plasma membrane 1 [Source:MGI Symbol;Acc:MGI:104653]	The protein encoded by this gene belongs to the family of P-type primary ion transport ATPases. These enzymes remove bivalent calcium ions from eukaryotic cells against very large concentration gradients.	Ion channel/binding	Enzyme	0.560473	2
Calml4	ENSMUSG00000032246	calmodulin-like 4 [Source:MGI Symbol;Acc:MGI:1922850]	Proteins are expected to exhibit calcium ion binding.	Ion channel/binding	Binding protein	1.710186	2
Car12	ENSMUSG00000032373	carbonic anhydrase 12 [Source:MGI Symbol;Acc:MGI:1923709]	Carbonic anhydrases are a large family of zinc metalloenzymes that catalyze the reversible hydration of carbon dioxide.	Hydrolase activity	Enzyme	1.997451	2
Cbr2	ENSMUSG00000025150	carbonyl reductase 2 [Source:MGI Symbol;Acc:MGI:107200]	May function in the pulmonary metabolism of endogenous carbonyl compounds, such as aliphatic aldehydes and ketones, 3-ketosteroids and fatty aldehydes, as well as in xenobiotic metabolism.	Oxidoreductase activity	Enzyme	-1.397167	1
Ccdc135	ENSMUSG00000031786	coiled-coil domain containing 135 [Source:MGI Symbol;Acc:MGI:2685616]	Function is yet unknown.	Enzyme regulator activity	Unknown	1.72537	2
Cdhr4	ENSMUSG00000032595	cadherin-related family member 4 [Source:MGI Symbol;Acc:MGI:1916648]	Function is yet unknown.	Others	Unknown	1.020248	2
Cdkn1c	ENSMUSG00000037664	cyclin-dependent kinase inhibitor 1C (P57) [Source:MGI Symbol;Acc:MGI:104564]	This gene is a potent tight-binding inhibitor of several G1 cyclin/CDK complexes (cyclin E-CDK2, cyclin D2-CDK4, and cyclin A-CDK2) and negative regulator of cell proliferation.	Cell cycle/development	Binding protein	0.666692	2
Chrna2	ENSMUSG00000022041	cholinergic receptor, nicotinic, alpha polypeptide 2 (neuronal) [Source:MGI Symbol;Acc:MGI:87886]	Nicotinic acetylcholine receptors are ligand-gated ion channels formed by a pentameric arrangement of alpha and beta subunits to create distinct muscle and neuronal receptors.	Ion channel/binding	Channel	-2.21204	1
Cldn2	ENSMUSG00000047230	claudin 2 [Source:MGI Symbol;Acc:MGI:1276110]	This gene product belongs to the claudin protein family whose members have been identified as major integral membrane proteins localized exclusively at tight junctions.	Cell adhesion/motility/cytoskeleton	Binding protein	3.317554	2
Clic6	ENSMUSG00000022949	chloride intracellular channel 6 [Source:MGI Symbol;Acc:MGI:2146607]	This gene encodes a member of the chloride intracellular channel family of proteins.	Ion channel/binding	Unknown	1.54298	2
Col16a1	ENSMUSG00000040690	collagen, type XVI, alpha 1 [Source:MGI Symbol;Acc:MGI:1095396]	This gene encodes the alpha chain of type XVI collagen, a member of the FACIT collagen family (fibril-associated collagens with interrupted helices) and serve to maintain the integrity of the extracellular matrix.	Structural constituent	Binding protein	-0.655148	1
Col18a1	ENSMUSG00000001435	collagen, type XVIII, alpha 1 [Source:MGI Symbol;Acc:MGI:88451]	This gene encodes the alpha chain of type XVIII collagen. This collagen is one of the multiplexins, extracellular matrix proteins that contain multiple triple-helix domains (collagenous domains).	Structural constituent	Receptor/ligand	-0.745678	1
Col27a1	ENSMUSG00000045672	collagen, type XXVII, alpha 1 [Source:MGI Symbol;Acc:MGI:2672118]	This gene plays a role during the calcification of cartilage and the transition of cartilage to bone.	Structural constituent	Binding protein	-1.069935	1
Col8a2	ENSMUSG00000056174	collagen, type VIII, alpha 2 [Source:MGI Symbol;Acc:MGI:88464]	This gene encodes the alpha 2 chain of type VIII collagen and is a major component of the basement membrane of the corneal endothelium and forms homo- or heterotrimers with alpha 1 (VIII) type collagens.	Structural constituent	Binding protein	1.070876	2
Col9a3	ENSMUSG00000027570	collagen, type IX, alpha 3 [Source:MGI Symbol;Acc:MGI:894686]	This gene encodes one of the three alpha chains of type IX collagen, the major collagen component of hyaline cartilage.	Structural constituent	Binding protein	0.599949	2
CR536618.1	ENSMUSG00000095562	No description	Function is yet unknown.	Unknown	Unknown	-0.578899	1
Cyp26b1	ENSMUSG00000063415	cytochrome P450, family 26, subfamily b, polypeptide 1 [Source:MGI Symbol;Acc:MGI:2176159]	This gene encodes a member of the cytochrome P450 superfamily. The cytochrome P450 proteins are monooxygenases which catalyze many reactions involved in drug metabolism and synthesis of lipids.	Oxidoreductase activity	Enzyme	-0.8726	1
Cyp2a5	ENSMUSG00000005547	cytochrome P450, family 2, subfamily a, polypeptide 5 [Source:MGI Symbol;Acc:MGI:88597]	This gene encodes a member of the cytochrome P450 superfamily. The cytochrome P450 proteins are monooxygenases which catalyze many reactions involved in drug metabolism and synthesis of lipids.	Oxidoreductase activity	Enzyme	-3.024675	1
Ddc	ENSMUSG00000020182	dopa decarboxylase [Source:MGI Symbol;Acc:MGI:94876]	The encoded protein catalyzes the decarboxylation of L-3,4-dihydroxyphenylalanine (DOPA) to dopamine, L-5-hydroxytryptophan to serotonin and L-tryptophan to tryptamine.	Dopaminergic phenotype	Enzyme	-0.540373	1
Dio2	ENSMUSG00000007682	deiodinase, iodothyronine, type II [Source:MGI Symbol;Acc:MGI:1338833]	This gene belongs to the iodothyronine deiodinase family. It activates thyroid hormone by converting the prohormone thyroxine by outer ring deiodination to bioactive 3,3',5-triiodothyronine (T3).	Hydrolase activity	Enzyme	0.88956	2
Dmrta2	ENSMUSG00000047143	doublesex and mab-3 related transcription factor like family A2 [Source:MGI Symbol;Acc:MGI:2653629]	Gene may be involved in sexual development.	Transcription/transcription factor activity	Unknown	0.950129	2
Ebf4	ENSMUSG00000053552	early B cell factor 4 [Source:MGI Symbol;Acc:MGI:2385972]	Gene belongs to the conserved Olf/EBF family of helix-loop-helix transcription factors, members of which play important roles in neural development and B-cell maturation.	Others	Transcription/translation/regulation	-0.568561	1
Eln	ENSMUSG00000029675	elastin [Source:MGI Symbol;Acc:MGI:95317]	Major structural protein of tissues such as aorta and nuchal ligament, which must expand rapidly and recover completely.	Structural constituent	Binding protein	-1.017741	1
Enpp2	ENSMUSG00000022425	ectonucleotide pyrophosphatase/phosphodiesterase 2 [Source:MGI Symbol;Acc:MGI:1321390]	This gene functions as both a phosphodiesterase, which cleaves phosphodiester bonds at the 5' end of oligonucleotides, and a phospholipase, which catalyzes production of lysophosphatidic acid.	Others	Enzyme	1.077356	2
Epha4	ENSMUSG00000026235	Eph receptor A4 [Source:MGI Symbol;Acc:MGI:98277]	This gene belongs to the ephrin (EPH) receptor subfamily of the protein-tyrosine kinase family. EPH and EPH-related receptors have been implicated in mediating developmental events, particularly nervous system.	Receptor activity	Receptor/ligand	0.585893	2
Eps8l1	ENSMUSG00000006154	EPS8-like 1 [Source:MGI Symbol;Acc:MGI:1914675]	This gene encodes a protein that is related to epidermal growth factor receptor pathway substrate 8 (EPS8), a substrate for the epidermal growth factor receptor.	Others	Unknown	-0.769885	1
Eps8l2	ENSMUSG00000025504	EPS8-like 2 [Source:MGI Symbol;Acc:MGI:2138828]	Gene may play a role in membrane ruffling and remodeling of the actin cytoskeleton.	Others	Binding protein	0.532316	2
F5	ENSMUSG00000026579	coagulation factor V [Source:MGI Symbol;Acc:MGI:88382]	This gene encodes an essential cofactor of the blood coagulation cascade. This factor circulates in plasma, and is converted to the active form by the release of the activation peptide by thrombin.	Transporter	Enzyme	3.178528	2
Fat2	ENSMUSG00000055333	FAT tumor suppressor homolog 2 (Drosophila) [Source:MGI Symbol;Acc:MGI:2685369]	This gene is the second identified human homolog of the Drosophila fat gene, which encodes a tumor suppressor essential for controlling cell proliferation during Drosophila development.	Cell cycle/development	Binding protein	-4.11405	1
Fetub	ENSMUSG00000022871	fetuin beta [Source:MGI Symbol;Acc:MGI:1890221]	This gene is a protease inhibitor required for egg fertilization and required to prevent premature zona pellucida hardening before fertilization.	Others	Binding protein	-3.16892	1
Folr1	ENSMUSG00000001827	folate receptor 1 (adult) [Source:MGI Symbol;Acc:MGI:95568]	Binds to folate and reduced folic acid derivatives and mediates delivery of 5-methyltetrahydrofolate to the interior of cells.	Transporter	Receptor/ligand	3.659537	2
Gabra4	ENSMUSG00000029211	gamma-aminobutyric acid (GABA) A receptor, subunit alpha 4 [Source:MGI Symbol;Acc:MGI:95616]	Gamma-aminobutyric acid (GABA) is the major inhibitory neurotransmitter in the mammalian brain where it acts at GABA-A receptors, which are ligand-gated chloride channels.	Ion channel/binding	Channel	0.945456	2
Gm15564	ENSMUSG00000086324	predicted gene 15564 [Source:MGI Symbol;Acc:MGI:3783013]	Function is yet unknown.	Unknown	Unknown	0.80603	2
Gm15996	ENSMUSG00000090026	predicted gene 15996 [Source:MGI Symbol;Acc:MGI:3802107]	Function is yet unknown.	Unknown	Unknown	-0.994912	1
Gm17167	ENSMUSG00000091542	predicted gene 17167 [Source:MGI Symbol;Acc:MGI:4937994]	Function is yet unknown.	Unknown	Unknown	-0.53065	1
Gm20481	ENSMUSG00000092609	predicted gene 20481 [Source:MGI Symbol;Acc:MGI:5141946]	Function is yet unknown.	Unknown	Unknown	0.845606	2
Gyltl1b	ENSMUSG00000040434	glycosyltransferase-like 1B [Source:MGI Symbol;Acc:MGI:2443769]	Glycosyltransferase which participates in glycosylation of alpha-dystroglycan and carry out the synthesis of glycoprotein and glycosphingolipid sugar chains.	Others	Enzyme	2.62719	2
Hcrt	ENSMUSG00000045471	hypocretin [Source:MGI Symbol;Acc:MGI:1202306]	hypocretin neuropeptide precursor of isoforms A and B,expressed in lateral hypothalamus and adjacent areas, potential mediator in the central feedback mechanism,that regulates feeding behavior.	Neuropeptide hormone activity	Receptor/ligand	2.316938	2
Hmgb2	ENSMUSG00000054717	high mobility group box 2 [Source:MGI Symbol;Acc:MGI:96157]	This gene encodes a member of the non-histone chromosomal high mobility group protein family. The proteins of this family are chromatin-associated and ubiquitously distributed in the nucleus.	Transcription/transcription factor activity	Transcription/translation/regulation	-0.92204	1
Hoxb3	ENSMUSG00000048763	homeobox B3 [Source:MGI Symbol;Acc:MGI:96184]	Sequence-specific transcription factor which is part of a developmental regulatory system that provides cells with specific positional identities on the anterior-posterior axis.	Transcription/transcription factor activity	Transcription/translation/regulation	-3.459729	1
Hoxb3os	ENSMUSG00000084844	homeobox B3, opposite strand transcript [Source:MGI Symbol;Acc:MGI:103211]	Function is yet unknown.	Unknown	Unknown	-3.464921	1
Hoxb5	ENSMUSG00000038700	homeobox B5 [Source:MGI Symbol;Acc:MGI:96186]	Sequence-specific transcription factor which is part of a developmental regulatory system that provides cells with specific positional identities on the anterior-posterior axis.	Transcription/transcription factor activity	Transcription/translation/regulation	-3.432353	1
Hoxc4	ENSMUSG00000075394	homeobox C4 [Source:MGI Symbol;Acc:MGI:96195]	This gene belongs to the homeobox family of genes. The homeobox genes encode a highly conserved family of transcription factors that play an important role in morphogenesis in all multicellular organisms.	Transcription/transcription factor activity	Transcription/translation/regulation	-3.517926	1
Hps1	ENSMUSG00000025188	Hermansky-Pudlak syndrome 1 homolog (human) [Source:MGI Symbol;Acc:MGI:2177763]	The gene encodes a protein for a component of multiple cytoplasmic organelles and is crucial for their normal development and function and intracellular protein sorting.	Others	Binding protein	-0.670962	1
Igfbp2	ENSMUSG00000039323	insulin-like growth factor binding protein 2 [Source:MGI Symbol;Acc:MGI:96437]	Insulin-like growth factor (IGF)-binding proteins (IGFBPs) are a family of six homologous proteins with high binding affinity for IGF-I and IGF-II and their main role is to modulate actions of free IGFs.	Others	Binding protein	0.80006	2
Igsf5	ENSMUSG00000000159	immunoglobulin superfamily, member 5 [Source:MGI Symbol;Acc:MGI:1919308]	Gene may function as a cell adhesion molecule rather than a tight-junction protein. It may participate in the adhesion between spermatogonia- spermatogonia, spermatogonia-Sertoli cells, and Sertoli cells.	Others	Binding protein	0.532068	2
Irx2	ENSMUSG00000001504	Iroquois related homeobox 2 (Drosophila) [Source:MGI Symbol;Acc:MGI:1197526]	IRX2 is a member of the Iroquois homeobox gene family. Members of this family appear to play multiple roles during pattern formation of vertebrate embryos.	Transcription/transcription factor activity	Transcription/translation/regulation	-1.142853	1
Iyd	ENSMUSG00000019762	iodotyrosine deiodinase [Source:MGI Symbol;Acc:MGI:1917587]	Catalyzes the oxidative NADPH-dependent deiodination of monoiodotyrosine (L-MIT) or diiodotyrosine (L-DIT) and acts during the hydrolysis of thyroglobulin to liberate iodide	Oxidoreductase activity	Enzyme	2.257859	2
Kcne2	ENSMUSG00000039672	potassium voltage-gated channel, Isk-related subfamily, gene 2 [Source:MGI Symbol;Acc:MGI:1891123]	Ancillary protein that assembles as a beta subunit with a voltage-gated potassium channel complex of pore-forming alpha subunits and modulates the gating kinetics and enhances stability of channel complex.	Ion channel/binding	Channel	5.383034	2
Kcnj13	ENSMUSG00000079436	potassium inwardly-rectifying channel, subfamily J, member 13 [Source:MGI Symbol;Acc:MGI:3781032]	This gene encodes a member of the inwardly rectifying potassium channel family of proteins. Members of this family form ion channel pores that allow potassium ions to pass into a cell.	Ion channel/binding	Channel	3.819521	2
Kitl	ENSMUSG00000019966	kit ligand [Source:MGI Symbol;Acc:MGI:96974]	This gene encodes the ligand of the tyrosine-kinase receptor encoded by the KIT locus. It acts in utero in germ cell and neural cell development, and hematopoiesis, all believed to reflect a role in cell migration.	Cell cycle/development	Receptor/ligand	0.864494	2
Kl	ENSMUSG00000058488	klotho [Source:MGI Symbol;Acc:MGI:1101771]	This gene encodes a type-I membrane protein that is related to beta-glucosidases.	Hydrolase activity	Enzyme	2.219972	2
Lama1	ENSMUSG00000032796	laminin, alpha 1 [Source:MGI Symbol;Acc:MGI:99892]	Binding to cells via a high affinity receptor, laminin is thought to mediate the attachment, migration and organization of cells into tissues during embryonic development.	Structural constituent	Receptor/ligand	-0.902549	1
Lars2	ENSMUSG00000035202	leucyl-tRNA synthetase, mitochondrial [Source:MGI Symbol;Acc:MGI:2142973]	This gene encodes a class 1 aminoacyl-tRNA synthetase, mitochondrial leucyl-tRNA synthetase. Each of the twenty aminoacyl-tRNA synthetases catalyzes the aminoacylation of a specific tRNA.	Others	Enzyme	0.660776	2
Lbp	ENSMUSG00000016024	lipopolysaccharide binding protein [Source:MGI Symbol;Acc:MGI:1098776]	The protein encoded by this gene binds to the lipid A moiety of bacterial lipopolysaccharides (LPS) and acts as an affinity enhancer for CD14, facilitating its association with LPS.	Others	Binding protein	0.895586	2
Lef1	ENSMUSG00000027985	lymphoid enhancer binding factor 1 [Source:MGI Symbol;Acc:MGI:96770]	Participates in the Wnt signaling pathway. Activates transcription of target genes in the presence of CTNNB1 and EP300. May play a role in hair cell differentiation and follicle morphogenesis.	Transcription/transcription factor activity	Transcription/translation/regulation	0.593499	2
Leng8	ENSMUSG00000035545	leukocyte receptor cluster (LRC) member 8 [Source:MGI Symbol;Acc:MGI:2142195]	Function not quite known yet.	Receptor activity	Unknown	-0.643245	1
Lrrtm1	ENSMUSG00000060780	leucine rich repeat transmembrane neuronal 1 [Source:MGI Symbol;Acc:MGI:2389173]	Exhibits strong synaptogenic activity, restricted to excitatory presynaptic differentiation, acting at both pre- and postsynaptic level.	Synaptic activity	Unknown	0.580738	2
Mab21l2	ENSMUSG00000057777	mab-21-like 2 (C. elegans) [Source:MGI Symbol;Acc:MGI:1346022]	This gene is similar to the C. elegans MAB-21 cell fate-determining gene, a downstream target of transforming growth factor-beta signaling. It is thought that this gene may be involved in neural development.	Others	Unknown	-1.811091	1
Mapk13	ENSMUSG00000004864	mitogen-activated protein kinase 13 [Source:MGI Symbol;Acc:MGI:1346864]	p38 MAP kinases act as an integration point for multiple biochemical signals and involved in a wide variety of cellular processes such as proliferation, differentiation, transcription regulation and development.	Enzyme regulator activity	Enzyme	-2.815269	1
Mas1	ENSMUSG00000068037	MAS1 oncogene [Source:MGI Symbol;Acc:MGI:96918]	This gene encodes a class I seven-transmembrane G-protein-coupled receptor. The encoded protein may play a role in multiple processes including hypotension, smooth muscle relaxation and cardioprotection.	Receptor activity	Receptor/ligand	1.504027	2
Mef2c	ENSMUSG00000005583	myocyte enhancer factor 2C [Source:MGI Symbol;Acc:MGI:99458]	The product of this gene is a member of the MADS/MEF2 family of DNA binding proteins. The protein is thought to regulate gene expression, including expression of the smooth muscle myosin heavy chain gene.	Transcription/transcription factor activity	Transcription/translation/regulation	0.524341	2
Meg3	ENSMUSG00000021268	maternally expressed 3 [Source:MGI Symbol;Acc:MGI:1202886]	This gene is a maternally expressed imprinted gene. Multiple alternatively spliced transcript variants have been transcribed from this gene and all of them are long non-coding RNAs.	Others	Transcription/translation/regulation	-0.557163	1
Mid1	ENSMUSG00000035299	midline 1 [Source:MGI Symbol;Acc:MGI:1100537]	The protein encoded by this gene is a member of the tripartite motif family and is likely involved in the formation of multiprotein structures acting as anchor points to microtubules.	Others	Binding protein	-0.509096	1
Mrap2	ENSMUSG00000042761	melanocortin 2 receptor accessory protein 2 [Source:MGI Symbol;Acc:MGI:3609239]	This gene may involve in type 1 melanocortin receptor binding and type 3 melanocortin receptor binding.	Others	Unknown	-0.699694	1
Msx1	ENSMUSG00000048450	homeobox, msh-like 1 [Source:MGI Symbol;Acc:MGI:97168]	This gene encodes a member of the muscle segment homeobox gene family. The encoded protein functions as a transcriptional repressor during embryogenesis.	Transcription/transcription factor activity	Transcription/translation/regulation	1.723836	2
Mt1	ENSMUSG00000031765	metallothionein 1 [Source:MGI Symbol;Acc:MGI:97171]	Metallothioneins have a high content of cysteine residues that bind various heavy metals; these proteins are transcriptionally regulated by both heavy metals and glucocorticoids.	Others	Binding protein	-0.457632	1
Mt2	ENSMUSG00000031762	metallothionein 2 [Source:MGI Symbol;Acc:MGI:97172]	Metallothioneins have a high content of cysteine residues that bind various heavy metals; these proteins are transcriptionally regulated by both heavy metals and glucocorticoids.	Others	Binding protein	-0.603719	1
Myo5b	ENSMUSG00000025885	myosin VB [Source:MGI Symbol;Acc:MGI:106598]	The protein encoded by this gene, together with other proteins, may be involved in plasma membrane recycling. Mutations in this gene are associated with microvillous inclusion disease.	Motor-related activity	Binding protein	0.618371	2
Neat1	ENSMUSG00000092274	nuclear paraspeckle assembly transcript 1 (non-protein coding) [Source:MGI Symbol;Acc:MGI:1914211]	Function is yet unknown.	Structural constituent	Unknown	-0.577607	1
Neurod6	ENSMUSG00000037984	neurogenic differentiation 6 [Source:MGI Symbol;Acc:MGI:106593]	Activates E box-dependent transcription in collaboration with TCF3/E47 and May be a trans-acting factor involved in the development and maintenance of the mammalian nervous system.	Cell cycle/development	Binding protein	1.500306	2
Ntng1	ENSMUSG00000059857	netrin G1 [Source:MGI Symbol;Acc:MGI:1934028]	Netrin G1 belongs to a conserved family of proteins that act as axon guidance cues during vertebrate nervous system development.	Others	Receptor/ligand	0.467996	2
Oca2	ENSMUSG00000030450	oculocutaneous albinism II [Source:MGI Symbol;Acc:MGI:97454]	The encoded protein is believed to be an integral membrane protein involved in small molecule transport, specifically tyrosine - a precursor of melanin.	Transporter	Transport	3.877846	2
Omp	ENSMUSG00000074006	olfactory marker protein [Source:MGI Symbol;Acc:MGI:97436]	Gene may act as a modulator of the olfactory signal- transduction cascade.	Others	Binding protein	-1.945394	1
Otp	ENSMUSG00000021685	orthopedia homolog (Drosophila) [Source:MGI Symbol;Acc:MGI:99835]	Gene encoding this protein may function during brain development and specifically required for the specification of diencephalic dopaminergic neurons of the A11 group.	Transcription/transcription factor activity	Binding protein	-1.118021	1
Otx2	ENSMUSG00000021848	orthodenticle homolog 2 (Drosophila) [Source:MGI Symbol;Acc:MGI:97451]	This gene encodes a member of the bicoid subfamily of homeodomain-containing transcription factors. The encoded protein acts as a transcription factor and plays a role in brain, craniofacial and development.	Transcription/transcription factor activity	Transcription/translation/regulation	0.75791	2
Oxt	ENSMUSG00000027301	oxytocin [Source:MGI Symbol;Acc:MGI:97453]	This gene encodes a precursor protein that is processed to produce oxytocin and neurophysin I. Oxytocin is posterior pituitary hormone which is synthesized in the system.	Neuropeptide hormone activity	Receptor/ligand	-1.278385	1
Pcp4	ENSMUSG00000090223	Purkinje cell protein 4 [Source:MGI Symbol;Acc:MGI:97509]	Gene may probably a regulator of calmodulin signaling.	Others	Binding protein	0.526223	2
Pdlim2	ENSMUSG00000022090	PDZ and LIM domain 2 [Source:MGI Symbol;Acc:MGI:2384850]	The encoded protein suppresses anchorage-independent growth and promotes cell migration and adhesion through interactions with the actin cytoskeleton via the PDZ domain.	Cell cycle/development	Binding protein	0.475867	2
Pdp1	ENSMUSG00000049225	pyruvate dehyrogenase phosphatase catalytic subunit 1 [Source:MGI Symbol;Acc:MGI:2685870]	Pyruvate dehydrogenase (E1) is one of the three components (E1, E2, and E3) of the large pyruvate dehydrogenase complex. Pyruvate dehydrogenase kinases catalyze phosphorylation of serine residues of E1.	Enzyme regulator activity	Enzyme	0.637417	2
Pitx2	ENSMUSG00000028023	paired-like homeodomain transcription factor 2 [Source:MGI Symbol;Acc:MGI:109340]	Gene controls cell proliferation in a tissue-specific manner and is involved in morphogenesis.	Transcription/transcription factor activity	Transcription/translation/regulation	0.919846	2
Pla2g5	ENSMUSG00000041193	phospholipase A2, group V [Source:MGI Symbol;Acc:MGI:101899]	This gene is a member of the secretory phospholipase A2 family. The encoded enzyme catalyzes the hydrolysis of membrane phospholipids to generate lysophospholipids and free fatty acids.	Others	Enzyme	1.486496	2
Plekhg1	ENSMUSG00000040624	pleckstrin homology domain containing, family G (with RhoGef domain) member 1 [Source:MGI Symbol;Acc:MGI:2676551]	Gene involves in guanyl-nucleotide exchange factor activity, Rho guanyl-nucleotide exchange factor activity.	Enzyme regulator activity	Transcription/translation/regulation	0.597129	2
Pmch	ENSMUSG00000035383	pro-melanin-concentrating hormone [Source:MGI Symbol;Acc:MGI:97629]	Gene may act as a neurotransmitter or neuromodulator in a broad array of neuronal functions directed toward the regulation of goal-directed behavior, such as food intake, and general arousal.	Neuropeptide hormone activity	Receptor/ligand	0.891733	2
Prg4	ENSMUSG00000006014	proteoglycan 4 (megakaryocyte stimulating factor, articular superficial zone protein) [Source:MGI Symbol;Acc:MGI:1891344]	The protein encoded by this gene is a large proteoglycan specifically synthesized by chondrocytes located at the surface of articular cartilage, and also by some synovial lining cells.	Others	Binding protein	-0.758414	1
Prkcd	ENSMUSG00000021948	protein kinase C, delta [Source:MGI Symbol;Acc:MGI:97598]	Protein kinase C (PKC) is a family of serine- and threonine-specific protein kinases. PKC family members phosphorylate a wide variety of protein targets and are involved in diverse cellular signaling pathways.	Enzyme regulator activity	Enzyme	1.149431	2
Prlr	ENSMUSG00000005268	prolactin receptor [Source:MGI Symbol;Acc:MGI:97763]	This is a receptor for the anterior pituitary hormone prolactin and Prolactin-dependent signaling occurs as the result of ligand-induced dimerization of the prolactin receptor.	Receptor activity	Receptor/ligand	0.971011	2
Ptk2b	ENSMUSG00000059456	PTK2 protein tyrosine kinase 2 beta [Source:MGI Symbol;Acc:MGI:104908]	This gene encodes a cytoplasmic protein tyrosine kinase which is involved in calcium-induced regulation of ion channels and activation of the map kinase signaling pathway.	Others	Enzyme	0.504718	2
Ptpn3	ENSMUSG00000038764	protein tyrosine phosphatase, non-receptor type 3 [Source:MGI Symbol;Acc:MGI:105307]	May act at junctions between the membrane and the cytoskeleton.	Cell adhesion/motility/cytoskeleton	Enzyme	0.667795	2
Ptpn4	ENSMUSG00000026384	protein tyrosine phosphatase, non-receptor type 4 [Source:MGI Symbol;Acc:MGI:1099792]	May act at junctions between the membrane and the cytoskeleton.	Cell adhesion/motility/cytoskeleton	Enzyme	0.688583	2
Ramp3	ENSMUSG00000041046	receptor (calcitonin) activity modifying protein 3 [Source:MGI Symbol;Acc:MGI:1860292]	The protein encoded by this gene is a member of receptor calcitonin activity modifying proteins and involves in transporting the calcitonin gene-related peptide type 1 receptor to the plasma membrane.	Receptor activity	Receptor/ligand	0.740486	2
Rasgrp1	ENSMUSG00000027347	RAS guanyl releasing protein 1 [Source:MGI Symbol;Acc:MGI:1314635]	This gene functions as a diacylglycerol-regulated nucleotide exchange factor specifically activating Ras through the exchange of bound GDP for GTP and activates the Erk/MAP kinase cascade.	Enzyme regulator activity	Transcription/translation/regulation	0.725169	2
Rbm47	ENSMUSG00000070780	RNA binding motif protein 47 [Source:MGI Symbol;Acc:MGI:2384294]	Gene may involve in nucleotide binding, RNA binding, and nucleic acid binding.	Transcription/transcription factor activity	Binding protein	1.226088	2
Rgs11	ENSMUSG00000024186	regulator of G-protein signaling 11 [Source:MGI Symbol;Acc:MGI:1354739]	This gene inhibits signal transduction by increasing the GTPase activity of G protein alpha subunits thereby driving them into their inactive GDP-bound form.	Enzyme regulator activity	Transcription/translation/regulation	-0.665402	1
Rgs16	ENSMUSG00000026475	regulator of G-protein signaling 16 [Source:MGI Symbol;Acc:MGI:108407]	This gene inhibits signal transduction by increasing the GTPase activity of G protein alpha subunits thereby driving them into their inactive GDP-bound form.	Enzyme regulator activity	Transcription/translation/regulation	0.562159	2
Rgs4	ENSMUSG00000038530	regulator of G-protein signaling 4 [Source:MGI Symbol;Acc:MGI:108409]	This gene inhibits signal transduction by increasing the GTPase activity of G protein alpha subunits thereby driving them into their inactive GDP-bound form.	Enzyme regulator activity	Transcription/translation/regulation	0.683003	2
Rora	ENSMUSG00000032238	RAR-related orphan receptor alpha [Source:MGI Symbol;Acc:MGI:104661]	The protein encoded by this gene is a member of the NR1 subfamily of nuclear hormone receptors and can bind to hormone response elements upstream of several genes to enhance the expression of those genes.	Transcription/transcription factor activity	Transcription/translation/regulation	0.63154	2
Rtel1	ENSMUSG00000038685	regulator of telomere elongation helicase 1 [Source:MGI Symbol;Acc:MGI:2139369]	ATP-dependent DNA helicase required to suppress inappropriate homologous recombination, thereby playing a central role DNA repair and in the maintenance of genomic stability.	Transcription/transcription factor activity	Enzyme	-0.5722	1
Sh3d19	ENSMUSG00000028082	SH3 domain protein D19 [Source:MGI Symbol;Acc:MGI:1350923]	Gene may be involved in suppression of Ras-induced cellular transformation and Ras-mediated activation of ELK1 and plays a role in the regulation of cell morphology and cytoskeletal organization.	Structural constituent	Protein	0.626049	2
Shank3	ENSMUSG00000022623	SH3/ankyrin domain gene 3 [Source:MGI Symbol;Acc:MGI:1930016]	Shank proteins are multidomain scaffold proteins of the postsynaptic density that connect moelcular cues that promote synapse formation and dendritic spine maturation.	Cell adhesion/motility/cytoskeleton	Binding protein	0.531843	2
Slc10a4	ENSMUSG00000029219	solute carrier family 10 (sodium/bile acid cotransporter family), member 4 [Source:MGI Symbol;Acc:MGI:3606480]	Function is yet unknown.	Transporter	Transport	-0.928745	1
Slc16a11	ENSMUSG00000040938	solute carrier family 16 (monocarboxylic acid transporters), member 11 [Source:MGI Symbol;Acc:MGI:2663709]	Proton-linked monocarboxylate transporter. Catalyzes the rapid transport across the plasma membrane of many monocarboxylates.	Transporter	Transport	-0.603183	1
Slc16a6	ENSMUSG00000041920	solute carrier family 16 (monocarboxylic acid transporters), member 6 [Source:MGI Symbol;Acc:MGI:2144585]	Proton-linked monocarboxylate transporter. Catalyzes the rapid transport across the plasma membrane of many monocarboxylates.	Transporter	Transport	-0.645428	1
Slc4a5	ENSMUSG00000068323	solute carrier family 4, sodium bicarbonate cotransporter, member 5 [Source:MGI Symbol;Acc:MGI:2443220]	This gene encodes a member of the sodium bicarbonate cotransporter (NBC) family, part of the bicarbonate transporter superfamily and are involved in intracellular pH regulation.	Transporter	Transport	2.904824	2
Slc6a20a	ENSMUSG00000036814	solute carrier family 6 (neurotransmitter transporter), member 20A [Source:MGI Symbol;Acc:MGI:2143217]	This gene is a member of the subgroup of transporter with unidentified substrates within the Na+ and Cl- coupled transporter family and mediates the calcium-dependent uptake of imino acid.	Transporter	Transport	-0.614218	1
Slc6a4	ENSMUSG00000020838	solute carrier family 6 (neurotransmitter transporter, serotonin), member 4 [Source:MGI Symbol;Acc:MGI:96285]	This gene encodes an integral membrane protein that transports the neurotransmitter serotonin from synaptic spaces into presynaptic neurons and terminates the action of serotonin and recycles it.	Transporter	Transport	-3.713175	1
Slco1a5	ENSMUSG00000063975	solute carrier organic anion transporter family, member 1a5 [Source:MGI Symbol;Acc:MGI:1351865]	Gene encodes protein to mediate the Na(+)-independent transport of organic anions such as taurocholate and thyroid hormones.	Transporter	Transport	2.0036	2
Sostdc1	ENSMUSG00000036169	sclerostin domain containing 1 [Source:MGI Symbol;Acc:MGI:1913292]	Gene may be involved in the onset of endometrial receptivity for implantation/sensitization for the decidual cell reaction and may enhance Wnt signaling and inhibits TGF-beta signaling.	Others	Binding protein	1.722189	2
Sphk1	ENSMUSG00000061878	sphingosine kinase 1 [Source:MGI Symbol;Acc:MGI:1316649]	This gene encodes a kinase that phosphorylates sphingosine into sphingosine-1-phosphate, which is involved in cell differentiation, motility, and apoptosis.	Cell adhesion/motility/cytoskeleton	Enzyme	-0.840375	1
Spp1	ENSMUSG00000029304	secreted phosphoprotein 1 [Source:MGI Symbol;Acc:MGI:98389]	The protein encoded by this gene is involved in the attachment of osteoclasts to the mineralized bone matrix. The encoded protein is secreted and binds hydroxyapatite with high affinity.	Others	Receptor/ligand	-0.812612	1
Stil	ENSMUSG00000028718	Scl/Tal1 interrupting locus [Source:MGI Symbol;Acc:MGI:107477]	Gene plays an important role in embryonic development as well as in cellular growth and proliferation; its long-term silencing affects cell survival and cell cycle distribution as well as decreases CDK1 activity.	Cell cycle/development	Binding protein	1.862967	2
Sulf1	ENSMUSG00000016918	sulfatase 1 [Source:MGI Symbol;Acc:MGI:2138563]	Gene exhibits arylsulfatase activity and highly specific endoglucosamine-6-sulfatase activity. It can remove sulfate from the C-6 position of glucosamine within specific subregions of intact heparin.	Hydrolase activity	Enzyme	0.7502	2
Synpo2	ENSMUSG00000050315	synaptopodin 2 [Source:MGI Symbol;Acc:MGI:2153070]	Gene has an actin-binding and actin-bundling activity.	Cell adhesion/motility/cytoskeleton	Binding protein	0.905304	2
Sytl4	ENSMUSG00000031255	synaptotagmin-like 4 [Source:MGI Symbol;Acc:MGI:1351606]	Gene encoding protein interacts with vesicles containing negatively charged phospholipids in a Ca(2+)-independent manner.	Synaptic activity	Binding protein	-0.583894	1
Th	ENSMUSG00000000214	tyrosine hydroxylase [Source:MGI Symbol;Acc:MGI:98735]	The protein encoded by this gene is involved in the conversion of tyrosine to dopamine. It is the rate-limiting enzyme in the synthesis of catecholamines, hence plays a key role in adrenergic neurons.	Dopaminergic phenotype	Enzyme	-0.673157	1
Thbs3	ENSMUSG00000028047	thrombospondin 3 [Source:MGI Symbol;Acc:MGI:98739]	The protein encoded by this gene belongs to the thrombospondin family. Thrombospondin family members are adhesive glycoproteins that mediate cell-to-cell and cell-to-matrix interactions.	Others	Binding protein	-0.663477	1
Tle2	ENSMUSG00000034771	transducin-like enhancer of split 2, homolog of Drosophila E(spl) [Source:MGI Symbol;Acc:MGI:104635]	Gro/TLE family proteins are required for many developmental processes, including lateral inhibition, segmentation, sex determination, dorsal/ventral pattern formation, and terminal pattern formation.	Cell cycle/development	Binding protein	-0.564693	1
Tmem38b	ENSMUSG00000028420	transmembrane protein 38B [Source:MGI Symbol;Acc:MGI:1098718]	This gene encodes an intracellular monovalent cation channel that functions in maintenance of intracellular calcium release.	Ion channel/binding	Binding protein	0.923091	2
Tmem72	ENSMUSG00000048108	transmembrane protein 72 [Source:MGI Symbol;Acc:MGI:2442707]	Function is yet unknown.	Others	Unknown	2.834523	2
Tmprss7	ENSMUSG00000033177	transmembrane serine protease 7 [Source:MGI Symbol;Acc:MGI:2686594]	Gene encoding serine protease which preferentially hydrolyzes peptides with Arg at the P1 position.	Receptor activity	Enzyme	-1.60791	1
Tnnt1	ENSMUSG00000064179	troponin T1, skeletal, slow [Source:MGI Symbol;Acc:MGI:1333868]	Troponin T is the tropomyosin-binding subunit of troponin, the thin filament regulatory complex which confers calcium-sensitivity to striated muscle actomyosin ATPase activity.	Motor-related activity	Binding protein	0.589565	2
Tnnt3	ENSMUSG00000061723	troponin T3, skeletal, fast [Source:MGI Symbol;Acc:MGI:109550]	Troponin T is the tropomyosin-binding subunit of troponin, the thin filament regulatory complex which confers calcium-sensitivity to striated muscle actomyosin ATPase activity.	Motor-related activity	Binding protein	-1.142869	1
Trpv4	ENSMUSG00000014158	transient receptor potential cation channel, subfamily V, member 4 [Source:MGI Symbol;Acc:MGI:1926945]	The encoded protein is a Ca2+-permeable, nonselective cation channel that is thought to be involved in the regulation of systemic osmotic pressure.	Ion channel/binding	Channel	1.465441	2
Ttll6	ENSMUSG00000038756	tubulin tyrosine ligase-like family, member 6 [Source:MGI Symbol;Acc:MGI:2683461]	This gene encode protein to mediate tubulin polyglutamylation in cilia and involved in the side-chain elongation step of the polyglutamylation reaction rather than in the initiation step.	Cell adhesion/motility/cytoskeleton	Enzyme	-1.7402	1
Ttr	ENSMUSG00000061808	transthyretin [Source:MGI Symbol;Acc:MGI:98865]	This gene encodes a carrier protein responsible for the transport of thyroid hormones and retinol.	Transporter	Binding protein	4.988116	2
Vav3	ENSMUSG00000033721	vav 3 oncogene [Source:MGI Symbol;Acc:MGI:1888518]	This gene encodes VAV proteins are guanine nucleotide exchange factors for Rho family GTPases that activate pathways leading to actin cytoskeletal rearrangements and transcriptional alterations.	Cell adhesion/motility/cytoskeleton	Transcription/translation/regulation	0.681998	2
Wdr86	ENSMUSG00000055235	WD repeat domain 86 [Source:MGI Symbol;Acc:MGI:1915466]	Function is yet unknown.	Others	Unknown	1.726446	2
Wfdc2	ENSMUSG00000017723	WAP four-disulfide core domain 2 [Source:MGI Symbol;Acc:MGI:1914951]	This gene encodes a protein that is a member of the WFDC domain family and contains eight cysteines forming four disulfide bonds at the core of the protein. It functions as a broad range protease inhibitor.	Others	Binding protein	3.341176	2

Supplementary Table 6: Enrichment analysis

FDR is controlled using Benjamini & Hochberg approach

	DAVID		MetaCore		
**GO Molecular Function**	**p-value**	**FDR**	**p-value**	**FDR**	**Genes**
calmodulin binding	2.97E-04	2.88E-03	1.952E-05	1.019E-02	Rgs11, Atp2b1, Sphk1, Pcp4, Adcy1, Trpv4, Myo5b, Rgs4
protein binding	3.61E-07	9.59E-05	1.469E-04	2.219E-02	Pdlim2, Cdkn1c, Ace, Rgs16, Neurod6, Sostdc1, Synpo2, Oca2, Prkcd, Hcrt, Tle2, Oxt, Igfbp2, Hps1, Atp2b1, Sphk1, Shank3, Leng8, Sh3d19, Ebf4, Tmprss7, Eps8l2, Kl, Aqp1, Cbr2, Sytl4, Dmrta2, Dio2, Th, Mapk13, Kcne2, Adamts10, Msx1, Ttll6, Clic6, Pdp1, Lbp, Mef2c, Hoxc4, Ddc, Cldn2, Epha4, Ttr, Mt2, Igsf5, Col16a1, Kitl, Tnnt1, Pmch, Eps8l1, Ramp3, Mid1, Ntng1, Stil, Pcp4, Lama1, Pla2g5, Ptk2b, Mrap2, Adcy1, Hmgb2, Col18a1, F5, Trpv4, Tnnt3, Prlr, Myo5b, Omp, Pitx2, 4930547N16Rik, Vav3, Spp1, Slc6a4, Rgs11, Otx2, Rgs4, Enpp2
transmembrane transporter activity	6.52E-12	4.11E-10	1.585E-04	2.219E-02	Tmem38b, Oca2, Slc16a6, Atp2b1, Abca4, Slc16a11, Slc4a5, Kcnj13, Aqp1, Kcne2, Clic6, Slc6a20, Gabra4, Slco1a5, Trpv4, Slc6a4, Chrna2, Ttr, Folr1,
transporter activity	1.25E-10	3.94E-09	1.700E-04	2.219E-02	Tmem38b, Oca2, Slc16a6, Atp2b1, Abca4, Slc16a11, Slc4a5, Kcnj13, Aqp1, Sytl4, Kcne2, Folr1, Clic6, Ramp3, Slc6a20, Gabra4, Slco1a5, Trpv4, Slc6a4, Chrna2
ion transmembrane transporter activity	5.89E-10	1.48E-08	2.921E-04	2.785E-02	Tmem38b, Oca2, Slc16a6, Atp2b1, Slc4a5, Kcnj13, Aqp1, Kcne2, Clic6, Slc6a20, Gabra4, Slco1a5, Trpv4, Slc6a4, Chrna2
substrate-specific transporter activity	8.78E-12	3.69E-10	3.222E-04	2.785E-02	Tmem38b, Oca2, Slc16a6, Atp2b1, Abca4, Slc4a5, Kcnj13, Aqp1, Kcne2, Clic6, Ramp3, Slc6a20, Gabra4, Slco1a5, Trpv4, Slc6a4, Chrna2
substrate-specific transmembrane transporter activity	1.79E-12	2.25E-10	5.954E-04	3.884E-02	Tmem38b, Oca2, Slc16a6, Atp2b1, Slc4a5, Kcnj13, Aqp1, Kcne2, Clic6, Slc6a20, Gabra4, Slco1a5, Trpv4, Slc6a4, Chrna2
receptor binding	2.03E-08	2.45E-06	6.696E-04	3.884E-02	Ace, Hcrt, Oxt, Shank3, Kl, Aqp1, Clic6, Lbp, Epha4, Ttr, Col16a1, Kitl, Pmch, Ramp3, Lama1, Pla2g5, Mrap2, Hmgb2, Myo5b, Vav3, Spp1

	DAVID		MetaCore		
**GO Biological Process**	**p-value**	**FDR**	**p-value**	**FDR**	**Genes**
ion transport/response to lithium ion	1.08E-08	3.44E-06	2.16E-08	2.40E-06	Abca4, Ace, Adcy1, Aqp1, Atp2b1, Car12, Chrna2, Clic6, Folr1, Gabra4, Hcrt, Igfbp2, Kcne2, Kcnj13, Oca2, Pla2g5, Prkcd, Ptk2b, Ramp3, Slc10a4, Slc16a6, Slc4a5, Slc6a20a, Slc6a4, Slco1a5, Tmem38b, Trpv4
cellular response to drug/toxin	4.02E-06	2.21E-04	1.09E-07	7.99E-05	Adcy1, Aqp1, Col18a1, Ddc, Hmgb2, Igfbp2, Kcne2, Kcnj13, Mas1, Mef2c, Mt1, Mt2, Pdp1, Prkcd, Ptk2b, Slc6a4, Th, Vav3
response to hormone	3.81E-02	3.86E-01	1.73E-07	8.46E-05	Ace, Dio2, Hmgb2, Igfbp2, Oxt, Spp1, Th
regulation of dendritic spine morphogenesis	Undetermined	Undetermined	5.48E-07	1.86E-04	Epha4, Shank3
forebrain development	2.51E-07	4.82E-05	2.58E-08	2.74E-06	Aqp1, Epha4, Lef1, Mas1, Mef2c, Msx1, Neurod6, Otp, Otx2, Pitx2, Shank3, Slc6a4, Stil, Th
memory	1.70E-02	9.71E-01	3.12E-06	4.57E-04	Chrna2, Oxt, Pla2g5, Shank3, Slc6a4, Th, Adcy1, Prkcd
regulation of muscle contraction	Undetermined	Undetermined	1.08E-05	1.21E-03	Chrna2, Prkcd, Tnnt1, Tnnt3
cartilage morphogenesis/development	1.59E-07	2.48E-05	1.48E-05	1.27E-03	Hoxb3, Hoxc4, Mef2c, Msx1
cell adhesion	1.86E-11	9.83E-09	3.35E-05	1.71E-03	Cdhr4, Cldn2, Col16a1, Col18a1, Col27a1, Epha4, F5, Fat2, Igsf5, Kitl, Lama1, Lef1, Pcp4, Prkcd, Ptk2b, Spp1, Sulf1, Thbs3
elevation of cytosolic calcium ion concentration	Undetermined	Undetermined	3.52E-05	1.71E-03	Chrna2, Hcrt, Mas1, Oxt, Pla2g5, Pmch, Ptk2b, Trpv4
germ cell/male gonad development	1.69E-06	9.62E-04	3.71E-05	1.71E-03	Acrbp, Col9a3, Hmgb2, Kitl, Mas1, Oca2, Pitx2
intracellular signal transduction	1.79E-12	5.76E-10	5.39E-05	2.03E-03	Adcy1, Cyp26b1, Eps8l1, Eps8l2, Igfbp2, Mapk13, Mt1, Mt2, Pla2g5, Prkcd, Prlr, Rasgrp1, Rgs11, Shank3, Sphk1, Vav3
nervous system development	5.16E-06	1.59E-03	1.89E-09	3.42E-07	Ace, Adcy1, Aqp1, Atp2b1, Cdkn1c, Chrna2, Col9a3, Dmrta2, Epha4, Gabra4, Hmgb2, Kcnj13, Lama1, Mab21l2, Mas1, Mef2c, Msx1, Mt2, Neurod6, Ntng1, Omp, Otp, Otx2, Pcp4, Pdp1, Pitx2, Pla2g5, Prkcd, Ptk2b, Shank3, Slc6a4, Sphk1, Spp1, Stil, Sulf1, Th, Trpv4

	DAVID		MetaCore		
**GO Localisation/Cellular Component**	**p-value**	**FDR**	**p-value**	**FDR**	**Genes**
cell periphery	3.59E-06	1.65E-04	4.749E-08	7.402E-06	Pdlim2, Ace, Rgs16, Kcnj13, Rasgrp1, Prkcd, Igfbp2, Hps1, Epha4, Slc16a6, Atp2b1, Abca4, Sphk1, Shank3, Slc16a11, Fat2, Sh3d19, Slc4a5, Tmprss7, Eps8l2, Kl, Aqp1, Sytl4, Dio2, Th, Kcne2, Folr1, Lrrtm1, Clic6, Mas1, Sulf1, Cldn2, Igsf5, Car12, Cdhr4, Kitl, Ramp3, Slc6a20a, Ntng1, 6330527O06Rik, Arrdc1, Pla2g5, Ptk2b, Mrap2, Gabra4, Adcy1, Slco1a5, F5, Trpv4, Prlr, Vav3, Slc6a4, Rgs11, Rgs4, Enpp2, Chrna2
plasma membrane	8.80E-09	8.01E-07	5.565E-08	7.402E-06	Ace, Rgs16, Rasgrp1, Prkcd, Igfbp2, Hps1, Epha4, Slc16a6, Atp2b1, Abca4, Sphk1, Shank3, Slc16a11, Fat2, Sh3d19, Slc4a5, Kcnj13, Tmprss7, Eps8l2, Kl, Aqp1, Sytl4, Dio2, Th, Kcne2, Folr1, Lrrtm1, Clic6, Mas1, Sulf1, Cldn2, Igsf5, Car12, Cdhr4, Kitl, Ramp3, Slc6a20a, Ntng1, 6330527O06Rik, Arrdc1, Pla2g5, Ptk2b, Mrap2, Gabra4, Adcy1, Slco1a5, F5, Trpv4, Prlr, Vav3, Slc6a4, Rgs11, Rgs4, Enpp2, Chrna2
extracellular region part	4.35E-04	2.49E-02	1.444E-06	1.280E-04	Ace, Sostdc1, Oxt, Igfbp2, Prg4, Kl, Aqp1, Col27a1, Col9a3, Adamts10, Fetub, Lbp, Sulf1, Ttr, COL16A1, Kitl, 1500015O10Rik, Pmch, Lama1, Hmgb2, Col18a1, Wfdc2, F5, Spp1, Enpp2
cell projection	9.72E-04	2.43E-02	2.950E-05	1.263E-03	Oxt, Atp2b1, Abca4, Sphk1, Shank3, Eps8l2, Aqp1, Th, Folr1, Ttll6, Lrrtm1, Ddc, Epha4, Eps8l1, 6330527O06Rik, Ptk2b, Ccdc135, Slco1a5, Trpv4, Myo5b, Omp, Spp1, Slc6a4, Rgs11, Otx2
extracellular space	5.42E-06	9.76E-05	3.283E-05	1.263E-03	Ace, Sostdc1, Oxt, Igfbp2, Prg4, Kl, Fetub, Lbp, Sulf1, Ttr, Kitl, 1500015O10Rik, Pmch, Lama1, Hmgb2, Col18a1, Wfdc2, F5, Spp1, Enpp2
synapse part	3.07E-05	2.05E-03	3.513E-05	1.263E-03	Hcrt, Oxt, Sphk1, Shank3, Sytl4, Th, Lrrtm1, Ddc, Epha4, Ptk2b, Gabra4, Chrna2
vesicle	4.65E-06	7.67E-05	9.954E-04	1.172E-02	Oca2, Hcrt, Oxt, Igfbp2, Hps1, Sphk1, Aqp1, Sytl4, Th, Acrbp, Ddc, 1500015O10Rik, 6330527O06Rik, Arrdc1, F5, Trpv4, Myo5b, Spp1

	DAVID		MetaCore		
**KEGG pathways**	**p-value**	**FDR**	**p-value**	**FDR**	**Genes**
mmu04912:GnRH signaling pathway	2.31E-05	9.25E-04			Adcy1, Ptk2b, Mapk13, Prkcd, Pla2g5
mmu04270:Vascular smooth muscle contraction	1.30E-03	1.29E-02			Ramp3, Adcy1, Prkcd, Pla2g5
mmu04062:Chemokine signaling pathway	4.29E-03	3.38E-02			Adcy1, Vav3, Ptk2b, Prkcd
mmu04510:Focal adhesion	5.44E-03	3.57E-02			Lama1, Vav3, Thbs3, Spp1
mmu04512:ECM-receptor interaction	1.04E-02	4.95E-02			Lama1, Thbs3, Spp1, Col27a1

	DAVID		MetaCore		
**MetaCore pathways**	**p-value**	**FDR**	**p-value**	**FDR**	**Genes**
Development_Spp1 signaling in osteoclasts			4.232E-04	4.322E-02	Ptk2b, Vav3, Spp1
Transcription_Role of the non-genomic action of Retinoic acid and phosphorylation of Retinoic acid			6.152E-04	4.322E-02	Prkcd, Mapk13, Ttr
G-protein signaling_Regulation of p38 and JNK signaling mediated by G-proteins			9.234E-04	4.322E-02	Mapk13, Mef2c, Ptk2b
Serotonin modulation of dopamine release			1.148E-03	4.322E-02	Prkcd, Adcy1, Slc6a4
Neurophysiological process_Dopamine D2 receptor signaling in CNS			1.594E-03	4.322E-02	Th, Ddc, Adcy1
Development_Thromboxane A2 pathway signaling			1.798E-03	4.322E-02	Mapk13, Prkcd, Adcy1
Development_A2B receptor: action via G-protein alpha s			1.906E-03	4.322E-02	Mapk13, Ptk2b, Adcy1
Immune response_IL-13 signaling via PI3K-ERK			1.906E-03	4.322E-02	Prkcd, Sphk1, Adcy1
Development_A1 receptor signaling			2.255E-03	4.322E-02	Prkcd, Mapk13, Adcy1
Development_Endothelin-1/EDNRA signaling			2.255E-03	4.322E-02	Prkcd, Adcy1, Ptk2b
Immune response _IFN gamma signaling pathway			2.379E-03	4.322E-02	Prkcd, Mapk13, Ptk2b
Neurophysiological process_Constitutive and activity-dependent synaptic AMPA receptor delivery			2.508E-03	4.322E-02	Shank3, Prkcd
Immune response_CCR5 signaling in macrophages and T lymphocytes			2.919E-03	4.752E-02	Mapk13, Ptk2b, Adcy1
